# Using Standardised International Oral Health-Related Datasets in 6 Countries

**DOI:** 10.1016/j.identj.2024.01.001

**Published:** 2024-02-02

**Authors:** Tom Broomhead, Rachael England, Stephen Mason, Michael Sereny, Sean Taylor, Georgios Tsakos, David Williams, Sarah R. Baker

**Affiliations:** a Unit of Oral Health, Dentistry and Society, School of Clinical Dentistry, University of Sheffield, Sheffield, UK; b FDI World Dental Federation, Geneva-Cointrin, Switzerland; c Haleon Research & Development (formerly GlaxoSmithKine Consumer Healthcare R&D), Weybridge, UK; d Private dental practice, Hannover, Germany; e Department of Epidemiology and Public Health, University College London, London, UK; f Centre for Dental Public Health and Primary Care, Faculty of Medicine and Dentistry, Queen Mary University of London, London, UK

**Keywords:** Global health, Oral health, Dental health surveys, Social determinants of health, Dental caries, Periodontal disease

## Abstract

**Introduction:**

Oral diseases affect a significant proportion of the world's population, yet international comparisons involving oral health outcomes have often been limited due to differences in the way country-level primary data are collected. In response to this, the World Dental Federation (FDI) Oral Health Observatory project was launched with the goal of collecting and producing standardised international data on oral health across countries. The aim of this descriptive cross-sectional study was to examine associations between self-reported general health and a range of factors (sociodemographics, oral health–related behaviours, oral impacts, clinical variables) using these standardised international datasets.

**Methods:**

Dentists within FDI member National Dental Associations who chose to take part in the project were selected using a multistage sampling method. The number of dentists in each cluster was set according to the proportion of the national population living in the area, and 50 patients per dentist were systematically approached to take part. Patients and dentists completed 2 separate questionnaires on a mobile app. Ordinal logistic regression (conducted in December 2022) was used to analyse the linked patient and dentist data from 6 countries: China (n = 2242); Colombia (n = 1029); India (n = 999); Italy (n = 711); Japan (n = 1271); and Lebanon (n = 798). Self-reported general health was the dependent variable, with age, sex, education, self-reported oral health–related behaviours, self-reported oral impacts, and clinical variables acting as the independent variables.

**Results:**

The results demonstrated a different pattern of associations in the different countries. Better self-reported general health was associated with degree-level education in all 6 countries and with reporting no oral impact and no sensitive teeth in 4 countries. Several country-specific patterns were also found, including the importance of tooth brushing in Colombia, periodontal health in Italy, and differing associations with sugary drinks consumption in India and Japan.

**Conclusions:**

These descriptive findings provide a basis for further research and, importantly, for advocacy in identifying patient oral health care needs according to both person-reported and clinical aspects. This can facilitate optimisation of service provision and potentially influence policy and investments.

## Introduction

Oral diseases represent a significant burden worldwide. As of 2010, around 3.5 billion people were reported to live with an oral health condition, including around 2 billion people experiencing untreated dental caries, the most prevalent noncommunicable disease globally. In addition, severe periodontitis was reported in around 1 billion people worldwide, with around 350 million cases of edentulism, and oral cancers were amongst the top 15 cancers worldwide.^1^ To date, there has been little international comparative research of oral health–related outcomes. Notable exceptions include comparisons of adult oral health–related behaviours^2^, ^3^, ^4^, ^5^ and dental attendance^6^ by welfare regimes using cross-national survey data. Cross-national comparisons of decayed, missing, and filled teeth and quality of life in children in 11 countries have also been carried out, examining associations with structural determinants such as macroeconomic policy, governance, and public and social policy.^7^ The WHO Global Oral Health Status report^1^ also makes global comparisons for oral health–related outcomes.

However, such comparisons of oral health–related outcomes or data sources between countries are rare and remain a challenge due to the high costs of conducting national oral health surveys and the complexity of coordinating standard approaches to surveys internationally.^8^^,^^9^ Previous research has therefore largely relied on secondary analysis of existing survey data or national samples, with all their concomitant limitations.^10^ As such, standardised, international primary data on oral conditions are needed to effectively evaluate and plan need-based oral health policies and services. Across countries, such data would allow for comparisons of the impact of different oral health policies and benchmarking of oral health and services for future advocacy purposes.^11^

To facilitate the collection of internationally standardised oral health data, the World Dental Federation (FDI) and the International Consortium for Health Outcomes Measurement (ICHOM) developed a set of oral health outcome measures, the FDI-ICHOM Adult Oral Health Standard Set (AOHSS), which covers a comprehensive spectrum of patient-centred oral health outcomes.^12^ In parallel, FDI established the Oral Health Observatory (OHO) project with the goal of collecting reliable, standardised oral health datasets for primary dental care internationally to help with advocacy at the national level and facilitate multination studies of oral health. The resulting OHO project data would, it was envisaged, enable evaluation of oral health care needs from the standpoint of both patients and dentists at national and global levels. The OHO project findings would then be used, via National Dental Associations (NDAs), to help plan and optimise service provision and influence policy and investment, which should over time lead to improved oral health outcomes.^13^

This descriptive cross-sectional study is the first to report on the standardised oral health data from the OHO project. Reporting on oral health–related factors, key sociodemographic variables, and their associations with self-reported general health was used as a test case for the OHO data in 6 countries: China, Colombia, India, Italy, Japan, and Lebanon. Whilst many studies to date have examined the impact of oral health on general and overall health outcomes^14^, ^15^, ^16^ and the importance of oral health–related quality of life as a component of general health,^17^ there have been no cross-nation comparative studies of oral health, oral health behaviours, and their association with general health using a standardised dataset.

## Methods

### Study design

This was a cross-sectional observational study of patients attending for general dental services in primary care. A mobile app containing 2 questionnaires, one completed by the patient and the other by the dentist about the patient's clinical oral health status, was used to generate data in dental practices. A third online questionnaire—not reported on here—collected information about the dental practices in which data were collected. Full details of the questionnaire development, NDA, and participant sampling and recruitment have been reported elsewhere,^18^ with brief details herein. The questionnaires can be found in the Supplementary Appendix.

### Country recruitment

Country recruitment for the OHO project began in May 2017. The project was open to all FDI member NDAs withthe capacity to implement the study protocol. Countries without active FDI member NDAs or where the NDAs were not willing or able to participate were ineligible. The data reported in this study are from the 6 countries most advanced with data collection as of July 2020, when the project was paused due to the COVID-19 pandemic. These countries were China, Colombia, India, Italy, Japan, and Lebanon.

### Dentist sampling and recruitment

A multistage sampling method was used to select dentists. Dentists registered with the NDA in the relevant country were clustered according to the primary administrative division (eg, state, province; this varied according to what was considered relevant for each country, and details of the included administrative divisions are available in Appendix 4) in which they were located. The number of dentists required in each cluster (primary administrative division) was set according to the proportion of the national population living there. Each NDA had access to a list of all practices and dentists for each cluster, and the required number of dentists per cluster was randomly selected by each NDA from those that expressed an interest to participate in the study.

### Patient sampling and recruitment

A modified systematic sampling method was used to sample participants amongst all patients attending the chosen dental clinic during the study period. There were no criteria for recruitment, and patients were randomly selected based on the day they attended the clinic. For example, one patient was surveyed each working day according to the order in which they arrived in practice; on the first day of the study the first patient was surveyed, on the second day of the study the second patient was surveyed, and so on. If the selected patient declined, the following patient was invited to participate. Fifty patients per dentist were surveyed, in line with recommendations from the World Health Organization's Oral Health Surveys Basic Methods (5th edition) document.^19^ To be eligible, patients had to be able to give informed consent and reside in the study country. Additional information on the sampling methods can be found in Appendix 5.

### Ethical approval

NDAs reviewed the project according to national regulations. In Japan and Lebanon, the project underwent ethical review and in both cases was approved (Niigata University Ethics Review Board, application 2017-0285; Lebanese Dental Association Ethics Review Board, application 54ETH/19). In China, Colombia, India, and Italy, the project was reviewed in line with national regulations, and as a result it was not necessary to submit to a separate ethical review body. Participating patients received the study information sheet from their participating dentist. Consent was obtained through the mobile app and had to be completed prior to the questionnaire. The patient information sheet and information pack provided to participating dentists can be found in the supplementary appendix.

### Data collection

Patients completed the patient questionnaire using a tablet computer in clinic waiting rooms prior to their appointment. Dentists completed the dentist questionnaire during the appointment or later using patient records. Data were encrypted when stored on the app and transferred to FDI's secure servers. The patient and dentist datasets were linked through participant IDs, allowing for the inclusion of clinical variables alongside the sociodemographic, oral health–related behaviours, and oral impact patient data.

### Variables: patient-reported

#### Sociodemographic variables

Three variables were included: age, sex, and education. Age was collected as a continuous variable and recategorised into 18 to 24, 25 to 44, 45 to 64, and 65+ years. Children were included in the OHO data collection but were excluded (under 18s) as part of this analysis. Sex was collected as female or male. Education was measured using the 10 response options from the International Standard Classification of Education^20^ recategorised into “university education,” “tertiary education” (postsecondary), “secondary education,” and “no education/primary education.”

#### Oral health–related behaviours

Three variables were included: brushing frequency, sugary drink consumption, and dental visiting. Brushing frequency was “2 or more per day,” “1 or more per day,” “weekly,” and “never.” Sugary drink consumption was “seldom or never,” “less than daily,” “several times a week to everyday,” and “multiple times a day.” Patient-reported dental visiting referred to the last time an individual visited their dentist. The categories were “less than a year ago,” “1 to 2 years ago,” “more than 2 years ago,” and “never.”

#### Self-reported oral impacts

Self-reported oral health was measured using 2 measures. The first was an oral impacts score that was created by combining 9 items related to impacts associated with the mouth, teeth, or dentures in the past 12 months: pain; discomfort; spitting or seeing blood when brushing; difficulty eating, chewing or biting food; difficulty speaking or pronouncing words; feeling embarrassed to smile or laugh; problems sleeping; limiting participation in social activities/difficulty enjoying contact with others; and difficulty carrying out work. Participants were asked to rate the extent to which these problems affected them on a scale from 0 (“not at all”) to 5 (“very much”). Responses of “no” to any of the items were given a score of 0. The scores for the 9 items were summed to give an overall impact score, with a potential range of 0 to 45. The second self-reported measure was the frequency of tooth sensitivity. This was categorised into the following 4 responses: “never,” “rarely,” “occasionally,” and “often.”

#### Self-reported general health

Self-reported general health was measured using the question, “How would you rate your general health?” Responses were on a 5-point Likert scale (very good to very poor). The variable was recategorised for the analysis into 3 categories: “very good/good,” “fair,” and “poor/very poor.”

#### Variables: dentist-reported

Three dentist-reported (clinical) measures were included: remaining number of teeth; number of teeth with caries; and periodontal status. Remaining number of teeth and teeth with caries were collected as continuous variables and recategorised for the analysis. Number of teeth were 32, 31 to 28, 27 to 24; and <24. Teeth with caries were 0, 1 to 5, 6 to 10, and >10. These categories were chosen based on the distribution of the clinical data across all countries. Periodontal status was collected as a categorical variable “healthy,” “gingivitis,” “pockets” (both shallow and deep), and “mobile teeth.” Full procedure for collecting the clinical data can be found in the supplementary appendix. The 3 clinical variables were based on the WHO Oral Health Surveys Basic Methods document.^19^ Dentists were instructed to follow this guidance, although no further formal training and calibration were conducted.

#### Data analysis

Ordinal logistic regression was used to assess associations between self-reported general health and the sociodemographic, oral health–related behaviours, self-reported oral impacts, and dentist-reported variables. The same analysis was carried out separately in each of the 6 countries, with the aim of exploring patterns separately in each of the 6 countries, in line with previous health-related research using international data and similar techniques, although not in an oral health context.^21^ Analyses were conducted using IBM SPSS Statistics (Version 28). All variables were coded so that the perceived “worst” outcome was given a higher value. Therefore, a positive association implied that as the score for one variable increased, the score for the other variable also increased, indicating a worse outcome.

## Results

There were 7049 participants across the 6 countries as follows: China (n = 2241); Colombia (n = 1029); India (n = 999); Italy (n = 711); Japan (n = 1271); and Lebanon (n = 798). Descriptive statistics for all variables are presented in Table 1. The lowest prevalence of participants reporting twice-a-day toothbrushing was in India (45.7%), where the highest prevalence of everyday sugary drink consumption (80.1%) was also reported. The greatest oral impact mean score (6.4) was found in Lebanon. The highest proportion of participants visiting the dentist in the past 12 months was in Japan (80.6%), and these participants had the lowest mean remaining number of teeth (23.7), lowest mean number of teeth with caries (1.9), and lowest prevalence of healthy periodontal status (20.6%). The country with the highest mean remaining number of teeth with caries was Lebanon (3.7), whose participants, interestingly, reported the highest percentage with very good or good general health ratings.

**Table 1:** Descriptive statistics across all study variables for each of the 6 countries: Lebanon, China, Italy, Colombia, Japan, and India.

	China	Colombia	India	Italy	Japan	Lebanon
**No.**	2242	1029	999	711	1271	798
**Mean age, y**	36.7	37.4	38.5	45.0	53.5	38.9
**SD**	14.2	15.6	14.5	16.8	17.3	15.1
**Range**	18–100	18–86	18–96	18–93	18–93	18–8
**Female, %**	42.1	47.6	38.3	57.2	56.9	50.4
**Education, degree and above, %**	54.2	23.3	32.6	19.8	36.4	56.0
**Toothbrushing twice a day, %**	78.0	81.8	45.7	79.9	81.7	61.4
**Sugary drinks everyday, %**	25.4	64.5	80.1	31.8	46.3	70.8
**Last visit to dentist in the last year, %**	51.6	53.4	52.1	67.1	80.6	65.5
**Healthy periodontal status, %**	37.1	52.2	53.0	43.0	20.6	43.1
**Mean oral impact score**	6.0	3.7⁎	5.6	5.6	4.4	6.4
**SD**	7.2	6.4	6.7	6.9	6.1	7.1
Range*	0–43	0–35	0–33	0–40	0–39	0–40
**Sensitive teeth, “often,” %**	11.5	7.1	16.6	12.9	5.5	5.0
**Mean No. of teeth**	27.3	26.8	25.0	23.9	23.7	25.9
**SD**	5.3	6.6	9.1	9.0	7.4	7.0
**Range**	0–32	0–32	0–32	0–32	0–32	0–32
**Mean No. of teeth with caries**	2.4	2.4	2.7	2.4	1.9	3.7
**SD**	3.3	2.6	3.9	4.6	4.5	4.5
**Range**	0–31	0–21	0–32	0–32	0–32	0–30
**General health rating, “very good/good,” %**	57.8	53.2	51.5	60.3	21.8	74.9

⁎ Colombia missing data on eating and social participation.

### Which oral health–related variables are associated with self-reported general health?

The results of the regression analyses for each of the 6 countries can be seen in Table 2. In China, education at degree (estimate = −1.11; 95% CIs, −0.60 to −1.63), tertiary (estimate = −0.73; 95% CIs, −0.20 to −1.26), and secondary levels (estimate = −0.54; 95% CIs = −0.04 to −1.04) were all significantly associated with self-reported general health (as education level increased, general health outcomes improved), as were sensitive teeth for those who experienced this never (estimate = −1.2; 95% CIs, −0.81 to −1.63) or rarely (estimate = −0.38; 95% CIs, −0.02 to −0.75) and those without oral impacts, that is, those who scored zero on the oral impact score (estimate = −0.54; 95% CIs, −0.03 to −1.05). Participants with no filled teeth reported a significant and positive association with self-reported general health (estimate = 1.61; 95% CIs, 0.08 to 3.14).

**Table 2:** Adjusted regression estimates and 95% confidence intervals for self-rated general health in each of the 6 countries.

	China (n = 2242)	Colombia (n = 1029)	India (n = 999)	Italy (n = 711)	Japan (n = 1271)	Lebanon (n = 798)
	Estimate (95% CIs)	Estimate (95% CIs)	Estimate (95% CIs)	Estimate (95% CIs)	Estimate (95% CIs)	Estimate (95% CIs)
**General health (ref = poor or very poor)**						
Good or very good	**−2.15 (−0.04 to −4.27)**	**−19.32 (−16.71 to −21.93)**	**−3.00 (−0.43 to −5.56)**	**−5.90 (−1.57 to −10.23)**	−3.03 (0.36 to −6.41)	−2.14 (1.80 to −6.07)
Fair	0.98 (3.09 to −1.13)	−**17.77 (**−**15.16 to** −**20.38)**	−0.98 (1.58 to −3.54)	−2.46 (1.83 to −6.74)	0.51 (3.89 to −2.87)	0.77 (4.70 to −3.16)
**Age, y (ref >65)**						
<25	−0.03 (0.52 to −0.59)	0.42 (1.24 to −0.41)	−0.20 (0.57 to −0.97)	**−2.00 (−1.04 to −2.96)**	−0.52 (0.21 to −1.24)	−**1.64 (**−**0.61 to** −**2.66)**
25–44	0.09 (0.60 to −0.42)	0.28 (1.02 to −0.47)	−0.22 (0.48 to −0.92)	**−1.20 (−0.56 to −1.84)**	0.11 (0.57 to −0.35)	−**1.89 (**−**1.03 to** −**2.75)**
45–64	−0.13 (0.36 to −0.61)	0.52 (1.19 to −0.15)	0.03 (0.72 to 0.66)	**−0.65 (−0.06 to −1.24)**	0.27 (0.63 to −0.10)	−**1.11 (**−**0.31 to** −**1.91)**
**Sex (ref = male)**						
Female	−0.15 (0.06 to −0.36)	−0.15 (0.18 to −0.49)	−0.21 (0.09 to −0.51)	−0.04 (0.35 to −0.42)	−0.22 (0.09 to −0.53)	0.29 (0.77 to −0.20)
**Education (ref = primary/no education)**						
Degree	**−1.11 (−0.60 to −1.63)**	**−1.10 (−0.50 to −1.71)**	**−0.54 (−0.09 to −0.99)**	**−0.95 (−0.06 to −1.83)**	**−2.03 (−0.98 to −3.08)**	**−0.83 (−0.03 to −1.63)**
Tertiary	**−0.73 (−0.20 to −1.26)**	**−1.14 (−0.05 to −1.78)**	−0.32 (0.24 to −0.88)	−**1.23 (**−**0.15 to** −**2.3)**	−**1.65 (**−**0.59 to** −**2.70)**	−0.55 (0.50 to −1.60)
Secondary	**−0.54 (−0.04 to −1.04)**	**−0.91 (−0.38 to −1.43)**	0.18 (0.62 to −0.26)	−0.40 (0.38 to −1.18)	−**1.65 (**−**0.61 to** −**2.69)**	−0.31 (0.46 to −1.07)
**Brushing (ref = never)**						
Twice or more per day	−0.94 (0.08 to −1.96)	−**19.40 (**−**18.29 to** −**20.50)**	0.01 (1.25 to −1.23)	0.97 (3.84 to −1.91)	1.20 (4.15 to −1.76)	−0.25 (1.62 to −2.12)
Once a day	−0.32 (0.72 to −1.36)	−**19.14 (**−**18.00 to** −**20.29)**	0.12 (1.35 to −1.12)	1.03 (3.93 to −1.87)	1.38 (4.33 to −1.57)	0.41 (2.29 to −1.48)
Less than daily	0.1 (1.29 to −1.09)	−18.86 (−18.86 to −18.86)	0.94 (2.50 to −0.61)	1.39 (4.45 to −1.67)	2.03 (5.25 to −1.19)	0.58 (2.54 to −1.39)
**Sugary drink consumption (ref = multiple times a day)**						
Seldom or never	0.04 (0.55 to −0.48)	−0.26 (0.48 to −1.00)	−0.40 (0.43 to −1.22)	−0.13 (0.60 to −0.85)	0.08 (0.55 to −0.40)	0.33 (1.40 to −0.74)
Less than daily	−0.24 (0.24 to −0.72)	−0.13 (0.45 to −0.71)	−0.25 (0.26 to −0.77)	−0.46 (0.44 to −1.35)	**0.52 (1.03 to 0.01)**	0.42 (1.46 to −0.61)
Several times a week to everyday	−0.3 (0.17 to −0.76)	−0.27 (0.12 to −0.66)	**−0.52 (−0.20 to −0.84)**	0.01 (0.41 to −0.39)	0.10 (0.48 to −0.29)	0.08 (0.61 to −0.45)
**Last dental visit (ref = never)**						
Less than a year ago	−0.22 (0.22 to −0.67)	0.06 (1.14 to −1.03)	0.26 (0.95 to −0.43)	−2.30 (0.16 to −4.76)	0.09 (0.68 to −0.50)	−0.04 (2.51 to −2.59)
1-2 years	−0.17 (0.31 to −0.64)	0.49 (1.56 to −0.58)	0.41 (1.13 to −0.30)	−2.12 (0.38 to −4.61)	0.07 (0.73 to −0.58)	0.17 (2.76 to −2.42)
2-3 years ago or more	0.08 (0.55 to −0.39)	0.17 (1.26 to −0.91)	0.04 (0.79 to −0.71)	−1.60 (0.89 to −4.09)	−0.01 (0.62 to −0.65)	0.28 (2.82 to −2.26)
**Sensitive teeth (ref = often)**						
Never	−**1.2 (**−**0.81 to** −**1.63**	0.46 (1.10 to −0.17)	**−1.66 (−1.21 to −2.12)**	**−1.13 (−0.47 to −1.80)**	**−0.88 (−0.19 to −1.58)**	−0.76 (0.16 to −1.69)
Rarely	**−0.38 (−0.02 to −0.75)**	0.36 (1.01 to −0.29)	**−0.60 (−0.16 to −1.04)**	−0.56 (0.03 to −1.16)	−0.39 (0.24 to −1.02)	−.60 (0.30 to −1.51)
Occasionally	−0.17 (0.17 to −0.50)	−0.25 (0.39 to −0.88)	**−0.58 (−0.18 to −0.98)**	−0.28 (0.29 to −0.85)	−0.23 (0.41 to −0.87)	−0.47 (0.42 to −1.35)
**QoL score (ref = >20)**						
0	**−0.54 (**−**0.03 to** −**1.05)**	−**1.59 (**−**0.73 to** −**2.45)**	−**1.03 (**−**0.22 to** −**1.83)**	**−1.28 (−0.30 to −2.27)**	−0.68 (0.19 to −1.55)	−0.86 (0.30 to −2.03)
1–10	−0.29 (0.19 to −0.77)	−**1.16 (**−**0.33 to** −**1.98)**	−0.28 (0.47 to −1.03)	−0.45 (0.46 to −1.37)	−0.00 (0.85 to −0.86)	−0.42 (0.50 to −1.34)
11–20	0.24 (0.74 to −0.27)	−0.39 (0.46 to −1.24)	−0.54 (0.22 to −1.29)	−0.20 (0.77 to −1.16)	0.90 (1.84 to −0.04)	0.30 (1.23 to −0.63)
**Teeth with caries (ref = >10)**						
0	−0.70 (0.16 to −1.55)	0.98 (2.54 to −0.58)	−0.70 (0.29 to −1.70)	−0.22 (0.73 to −1.18)	0.15 (0.95 to 0.65)	0.62 (1.72 to −0.48)
1–5	−0.56 (0.27 to −1.40)	1.21 (2.73 to −0.30)	−0.32 (0.63 to −1.28)	−0.02 (0.94 to −0.97)	0.23 (1.05 to −0.60)	0.62 (1.67 to −0.42)
6–10	0.13 (1.04 to −0.78)	1.52 (3.10 to −0.06)	−0.72 (0.32 to −1.76)	−0.31 (0.83 to −1.45)	−0.08 (1.00 to −1.17)	0.81 (1.92 to −0.30)
**Missing teeth (ref = >10)**						
0	−0.56 (0.08 to −1.20)	−0.57 (0.83 to −1.96)	−0.09 (0.60 to −0.79)	−0.27 (0.78 to −1.33)	0.08 (0.88 to −0.71)	−0.00 (1.88 to −1.89)
1–5	−0.49 (0.12 to −1.10)	−0.08 (0.89 to −1.06)	0.02 (0.67 to −0.63)	−0.42 (0.32 to −1.16)	−0.06 (0.58 to −0.70)	−1.31 (0.12 to −2.73)
6–10	−0.21 (0.41 to −0.84)	0.76 (1.53 to −0.02)	−0.08 (0.74 to −0.09)	−0.19 (0.53 to −0.90)	−0.08 (0.47 to −0.64)	−**1.20 (**−**0.04 to** −**2.36)**
**Filled teeth (ref = >10)**						
0	**1.61 (3.14 to 0.08)**	0.37 (1.08 to −0.35)	0.43 (2.16 to −1.31)	0.27 (1.01 to −0.47)	−0.41 (0.19 to −1.01)	−0.03 (0.94 to −1.01)
1–5	1.36 (2.88 to −0.17)	0.19 (0.77 to −0.38)	0.54 (2.26 to −1.19)	0.17 (0.76 to −0.42)	**−0.40 (−0.04 to −0.77)**	0.19 (0.88 to −0.51)
6–10	1.07 (2.63 to −0.49)	−0.39 (0.22 to −1.00)	−0.26 (1.61 to −2.12)	0.30 (0.92 to −0.33)	0.060 (0.41 to −0.29)	−0.16 (0.54 to −0.86)
**No. of teeth (ref = <24)**						
32	−0.14 (0.45 to −0.73)	1.20 (2.58 to −0.19)	−0.68 (0.01 to 1.36)	−0.29 (0.81 to −1.39)	−0.14 (0.84 to −1.11)	−0.56 (1.33 to −2.44)
28–31	−0.34 (0.18 to −0.86)	0.30 (1.23 to −0.64)	**−0.75 (−0.25 to −1.26)**	0.14 (0.83 to −0.56)	−0.25 (0.34 to −0.83)	0.19 (1.57 to −1.20)
24–27	−0.31 (0.21 to −0.82)	−0.18 (0.56 to −0.92)	−0.53 (0.15 to −1.20)	−0.07 (0.59 to −0.73)	−0.11 (0.39 to −0.62)	0.77 (1.92 to −0.39)
**Periodontal status (ref = mobile teeth)**						
Healthy	0.11 (0.77 to −0.55)	−1.14 (0.21 to −2. 49)	−0.83 (0.03 to −1.70)	**−2.22 (−0.76 to −3.69)**	−0.89 (0.2 to −1.81)	−1.32 (0.84 to −3.48)
Gingivitis	0.06 (0.70 to −0.59)	−0.25 (1.07 to −1.57)	−0.59 (0.31 to −1.49)	**−2.49 (−1.01 to −3.97)**	−0.77 (0.14 to −1.68)	−0.59 (1.54 to −2.72)
Pockets	0.32 (0.96 to −0.32)	0.48 (1.81 to −0.85)	−0.61 (0.29 to −1.51)	**−2.29 (−0.84 to −3.74)**	−0.50 (0.37 to −1.38)	−0.42 (1.71 to −2.56)

Significant associations are in bold.

QoL, Quality of life.

In Colombia, those with degree (estimate = −1.10; 95% CIs, −0.50 to −1.71), tertiary (estimate = −1.14; 95% CIs, −0.50 to −1.78), and secondary levels of education (estimate = −0.91; 95% CIs, −0.38 to −1.43) had better self-related general health than those with primary/no education. Brushing teeth twice a day or more (estimate = −19.40; 95% CIs, −18.29 to −20.50) or once a day (estimate = −19.14; 95% CIs, −18.00 to −20.29) was associated with better general health, as was reporting oral impact scores of zero (estimate = −1.59; 95% CIs, −0.73 to −2.45) or 1 to 10 (estimate = −1.16; 95% CIs, −0.33 to −1.98).

In India, those with degree-level education reported better general health (estimate = −0.54; 95% CIs, −0.09 to −0.99), as did those who consumed sugary drinks “several times a week to everyday” (estimate = −0.52; 95% CIs, −0.20 to −0.84) compared to multiple times a day. Similarly, participants who never experienced sensitive teeth (estimate = −1.66; 95% CIs, −1.21 to −2.12) as well as those experiencing this rarely (estimate = −0.60; 95% CIs, −0.16 to −1.04) and occasionally (estimate = −0.58; 95% CIs, −0.18 to −0.98) reported better general health than those who often had sensitive teeth. This was also the case for participants with no oral impacts (estimate = −1.03; 95% CIs, −0.22 to −1.83) as well as participants with 28 to 31 teeth in their mouth (estimate = −0.75; 95% CIs, −0.25 to −1.26), that is, better self-reported general health.

In Italy, age was significantly associated with self-reported general health such that those aged younger than 25 (estimate = −2.00; 95% CIs, −1.04 to −2.96), 25 to 44 (estimate = −1.20; 95% CIs, −0.56 to −1.84), and 45 to 64 (estimate = −0.65; 95% CIs, −0.06 to −1.24) had better general health than those aged 65+ years. This was also the case for those educated to degree (estimate = −0.95; 95% CIs, −0.06 to −1.83) and tertiary levels (estimate = −1.23; 95% CIs, −0.15 to −2.31). Again, those who never experienced sensitive teeth and those who scored zero for the oral impact score reported better self-reported general health (estimate = −1.13; 95% CIs, −0.47 to −1.80 and estimate = −1.28; 95% CIs = −0.30 to −2.27, respectively). Italian participants with healthy gums (estimate = −2.22; 95% CIs, −0.76 to −3.69), gingivitis (estimate = −2.49; 95% CIs, −1.01 to −3.97), and periodontal pockets (estimate = −2.29; 95% CIs, −0.84 to −3.74) had better general health ratings than those with mobile teeth.

In Japan, those with degree (estimate = −2.03; 95% CIs, −0.98 to −3.08), tertiary (estimate = −1.65; 95% CIs, −0.59 to −2.70), and secondary levels of education (estimate = −1.65; 95% CIs, −0.61 to −2.69), as well as participants who never experienced sensitive teeth (estimate = −0.88; 95% CIs, −0.19 to −1.58) and participants with 1 to 5 filled teeth (estimate = −0.40; 95% CIs, −0.04 to −0.77), reported higher general health ratings. Japanese participants who consumed sugary drinks “less than daily” (estimate = 0.52; 95% CIs, 0.01 to 1.03) had better general health than those consuming sugary drinks multiple times a day.

Finally, in Lebanon, those participants aged younger than 25 (estimate = −1.64; 95% CIs, −0.61 to −2.66), 25 to 44 (estimate = −1.89; 95% CIs, −1.03 to −2.75), and 45 to 64 years (estimate = −1.11; 95% CIs, −0.31 to −1.91) had better self-reported general health than those aged 65+ years. Degree-level education (estimate = −0.83; 95% CIs, −0.03 to −1.63) and having 6 to 10 missing teeth (estimate = −1.20; 95% CIs, −0.04 to −2.36) was also associated with higher ratings of general health.

## Discussion

### Summary of findings

Degree-level education was associated with better self-reported general health in all 6 countries, with tertiary and secondary education also being associated with better health in 3 countries (China, Colombia, and Japan) compared to participants with primary or no formal education. Participants who never experienced sensitive teeth were also more likely to report better general health in China, India, Italy, and Japan, as were those who reported no oral impacts in China, Colombia, India, and Italy. Younger age categories were also associated with better self-reported general health in Japan and Lebanon.

Associations between more frequent brushing and better self-reported general health were found in Colombia only. Sugary drink consumption was associated with self-reported general health amongst Indian and Japanese participants, although in different directions (negative associations in India and positive associations in Japan). Periodontal health was associated with better self-reported general health in Italy but not in the remaining 5 countries.

### Interpretation

This descriptive analysis has demonstrated the different patterns by which oral health is associated with self-reported general health. Some of the findings are in line with previous literature, including the importance of age for general health^22^—despite some contradictory results on self-rated health^23^^,^^24^—and the influence of sociodemographic variables such as education.^25^ However, other key oral health–related variables such as tooth brushing^26^ and sugary drink consumption^27^ were, somewhat surprisingly based on current literature, not prominent in this descriptive analysis.

The frequent appearance of the oral impact score as a key variable demonstrates the importance of person-reported measures related to the everyday impacts of oral health, including tasks and situations not associated with oral health–related practices (eg, work, socialising). Some counterintuitive results were also found, such as having no filled teeth being associated with worse self-reported general health in China as well as participants in Lebanon with 6 to 10 missing teeth being more likely to report better general health compared to other Lebanese participants with different numbers of missing teeth. These cross-sectional associations may be due to the direct (or indirect) role of other variables not included in the OHO dataset. It is important to note that the analysis reported here can only be exploratory, and any interpretation of the data is complicated by the differing contexts of the countries involved. It is vitally important to consider macro-level differences between countries, including health care systems and differing sociocultural practices (and networks) and structural policies, which have previously been shown to be important for oral health.^7^^,^^28^, ^29^ Future follow-up OHO data are required together with hypothesis generation and comparative causal modelling to understand the patterns occurring in each country, and at a regional level, in greater depth. Such contextual research may also help in the interpretation of some of the more counterintuitive findings seen here. Nevertheless, the present descriptive findings can help to aid the primary goals of the OHO project, that is, to evaluate the needs of patients and dentists to improve service provision and in the use of data to identify health trends and influence policy by providing insight into the oral health and related factors of dental practice attenders. Data on populations that attend dental practices can also be of value for future planning of service provision.

### Strengths and limitations

Strengths of the analysis include the use of a standardised and comparable international dataset, allowing a rare opportunity to analyse key variables across multiple countries from South America, the Middle East, South Asia, and East Asia that are underrepresented in the oral health literature to date. The questionnaires (and resulting dataset) were based on important sociodemographic variables and oral health–related behaviours identified as important within the literature on oral and general health.

There were also limitations. Despite using standardised data, missing oral impact data in Colombia may have affected the analysis of oral impacts in this country. Additionally, due to the data being collected in dental practices, conclusions can only be drawn about those who attend the dentist and the sample may overrepresent groups with certain characteristics, as the datasets are not representative at a national level. The sample was also recruited from practices that are members of their NDA (non-NDA members were not included in the data collection), which again may not be representative of all practices. Additionally, the way in which clinical variables (remaining number of teeth, number of teeth with caries, periodontal status) were categorised (in order to be included in statistical analysis and match the format of other variables) may have led to a loss of information and a reduction in sensitivity in the variables. There was also no formal training or calibration of the clinical data collection to assure uniform interpretation of the clinical criteria. However, the collected data reflected a more pragmatic approach about the way oral diseases are reported in the different contexts involved in this project, and this has value in itself. Lack of use of a conceptual model to select variables for the analysis is another limitation.

### Future work

The OHO project is beneficial in that it has the potential to deliver real-time oral health–related data from several different settings. National oral health surveys, while still vital resources for understanding the state of oral health in a given country and of the trends in oral health when repeated over time, often have excessive time intervals (sometimes up to a decade) between consecutive surveys/data collections, meaning that the data can become less relevant over time as population patterns change. The OHO offers a great deal of information (along with valuable covariates) on a variety of subject-specific outcomes. Attempts to make future data representative and to reach groups who are typically not captured by this type of data collection would be beneficial, as could the use of existing methods to re-weight the data into representative samples. Additionally, subnational sample sizes in each country are relatively small, which could affect further regional analysis of oral health–related factors.

Following the COVID-19 pandemic, the OHO project has started data collection in 2 additional countries, with the aim to include more countries in the future. Due to the way data are collected (via a mobile app), the project has great potential to act as a national surveillance system for oral health, at least amongst those attending dental services, if distributed more widely within participating countries. This would also aid in the generation of real-time evidence, which so often is not available in large samples at the population level within dental and oral health research. Workshops have also been taking place with NDAs in participating countries, with the OHO data contributing to advocacy-related discussions. These workshops have already started to produce promising outcomes, including the introduction of national social marketing campaigns, an annual “no sugar day,” and other health-related policies in India.^30^ It is hoped that future work with the OHO data and participating NDAs could further contribute to beneficial action. The data are also well suited to multilevel modelling (patients clustered within dental practices within countries), and future work will look to apply this and other more complex methods to the data.

This paper, coauthored by the OHO overall team, presents a descriptive overview of associations using a standardised data collection method and tool across countries. As such, it was felt to be inappropriate to comment on, or speculate about, the potential contribution of contextual factors, health care systems, and other characteristics of the participating countries that might have influenced the results. Future work will therefore also include discussions with participating NDAs as well as other collaborators in these countries which are in a better position to comment on these factors, and this would add valuable context to the data and results moving forward. The data are already being taken forwards in some of the participating countries (China and Japan),^31^^,^^32^ with others in preparation to do so (India). These contributions may also allow for the direct comparison of patterns between countries if the proper contextual factors can be accounted for.

## Conclusions

This was the first study to use internationally standardised oral health data to analyse associations amongst sociodemographics, oral health-related variables, and self-reported general health. The findings demonstrate the pattern of associations that are important (and different) for general health outcomes in each country. These data have great potential and can act as a starting point for further research as well as for advocacy, identifying oral health care needs of patients from both person-reported and clinical standpoints. This can help optimise service provision and influence policy and investments as part of the OHO project aims. Further research into country-specific patterns and investigations at subnational geographies may provide additional contextual information alongside this analysis.

## Conflict of interest

None disclosed.

## Appendix Supplementary materials

Image, application 1

Image, application 2

Image, application 3

## Footnotes

Supplementary material associated with this article can be found in the online version at doi:10.1016/j.identj.2024.01.001.
