# Supplementary material for: Using Standardised International Oral Health-Related Datasets in 6 Countries
Source: Int Dent J. 2024 Feb 2;74(3):647–55. doi: 10.1016/j.identj.2024.01.001 (PMC11123530; doi:10.1016/j.identj.2024.01.001)
Supplement: Supplementary file 1 [file mmc1.docx]

Using standardised international oral health-related datasets in six countries

Broomhead T^1^, England R^2^, Mason S^3^, Sereny M^4^, Taylor S^2^, Tsakos G^5^, Williams DM^6^, Baker SR^1*^

Supplementary Appendices

Appendix 1 Patient Information Sheet

Appendix 2 Questionnaires

Appendix 3 Dentist Information Kit

Appendix 1 Patient Information Sheet


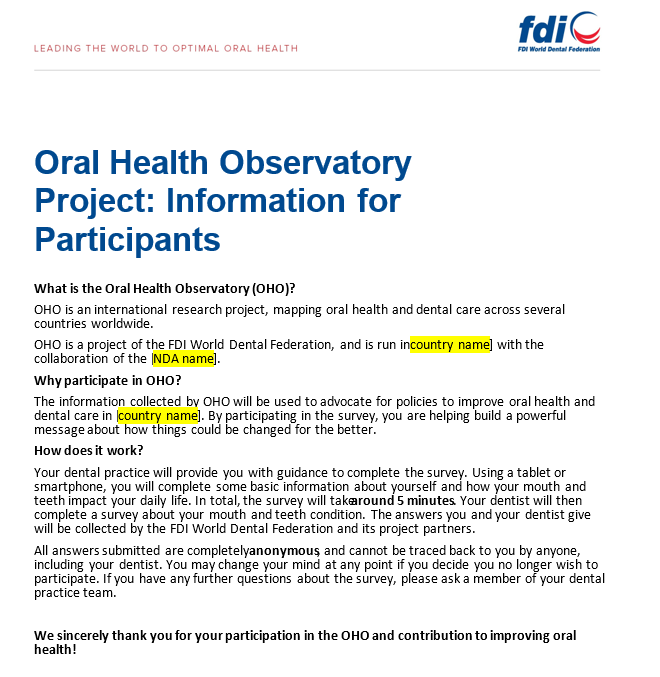


Appendix 2 Questionnaires (and response options)

* Denotes optional question, included at discretion of NDA

^All oral impact questions include severity rating on a scale of 1 to 5 for patients indicating that they have experienced that impact

| **Patient Questionnaire** |
| --- |
| **What is your age?**  *Continuous* |
| **What is your sex?**   - *Male* - *Female* - *[Locally appropriate options as determined by NDA]* - *Prefer not to say* |
| **What is the highest level of education you have completed or the highest degree you have achieved?**   - *No formal education* - *Early childhood education* - *Primary Education* - *Lower secondary education* - *Upper secondary education* - *Post-secondary non-tertiary education* - *Short-cycle tertiary education* - *Bachelor’s or equivalent level* - *Master’s or equivalent level* - *Doctoral or equivalent level* - *Rather not answer* |
| **What is your ethnicity?***  *Locally appropriate answer options as determined by NDA* |
| **When did you last visit the dentist?**   - *Less than 1 year* - *1-2 years* - *2-3 years* - *More than 3 years* - *Never* - *Don’t know/not sure* - *Rather not answer* |
| **[If patient has previously visited the dentist]**  **What was the purpose of the visit?**   - *Check-up* - *Routine treatment* - *Emergency treatment* - *Don’t know/not sure* - *Rather not answer* |
| **[If patient has not visited the dentist in the past year]**  **What were the reasons for not visiting the dentist sooner (pick all that apply)?**   - *Too busy* - *Dental office too far away* - *Nothing wrong with teeth* - *Afraid/don’t like dentists or dental treatment* - *Dental problems not serious enough* - *Could not get an appointment* - *Lack of or insufficient reimbursement* - *Dentist opening hours inconvenient with personal schedule* - *Other* - *Don’t know/not sure* - *Rather not answer* |
| **[If patient has previously visited the dentist]**  **How long does it take to travel to the dental office?**   - *Less than 30 minutes* - *30 minutes to 1 hour* - *1 to 3 hours* - *More than 3 hours* - *Don’t know/not sure* - *Rather not answer* |
| **[If patient has previously visited the dentist]**  **If you need dental care, you usually get an appointment within…**   - *24 hours* - *1 to 3 days* - *3 to 7 days* - *1 to 3 weeks* - *More than 3 weeks* - *Don’t know/not sure* - *Rather not answer* |
| **Do you currently have dental insurance for [current year]?***   - *Yes* - *No* - *Don’t know/not sure* - *Rather not answer* |
| **[If patient has previously visited the dentist]**  **Have you ever had a discussion with a dental professional on any of the following (pick all that apply)?**   - *Sensitivity management* - *Bleeding gums* - *Periodontal disease* - *Denture care* - *No, not necessary given condition* - *Rather not answer* |
| **How often do you brush your teeth or dentures?**   - *2 or more times per day* - *1 time per day* - *A few times per week* - *1 time per week* - *Never* - *Not applicable* - *Don’t know/not sure* - *Rather not answer* |
| **[If patient brushes teeth or dentures]**  **When (pick all that apply)?**   - *Morning before breakfast* - *Morning after breakfast* - *After lunch* - *Before going to sleep* - *Other time of day* - *Don’t know/not sure* - *Rather not answer* |
| **Do you use any of the following fluoride products (pick all that apply)?**   - *Toothpaste* - *Tablets/drops* - *Salt* - *Mouth rinse* - *Other* - *Don’t know/not sure* - *Rather not answer* |
| **Do you wear a denture or removable appliance?**   - *No* - *I wear a full upper denture* - *I wear a full lower denture* - *I wear a partial upper denture* - *I wear a partial lower denture* - *I wear an ortho retainer* - *I wear a mouthguard* - *I wear a removable orthodontic appliance* - *Rather not answer* |
| **[If patient wears a denture or removable appliance]**  **Have you used a fixative for your denture or removable appliance?**   - *No* - *Yes, less than once a month* - *Yes, once a month* - *Yes, once a day* - *Yes, overnight* - *Yes, during the day* - *Yes, mor than once a day* - *Rather not answer* |
| **[If patient wears a denture or removable appliance]**  **Have you do you use a specialized cleanser for your denture or removable appliance?**   - *Overnight* - *During the day* - *More than once a day* - *Once a day* - *Once a week* - *Once a month* - *Less than once a month* - *Never* - *Rather not answer* |
| **How often do you eat any of the following foods, even in small quantities…**  **Biscuits, cakes, buns, sweets/candy, jam or honey, chewing gum with sugar [plus any locally appropriate options set by NDA]?**   - *4 or more times a day* - *2-3 times a day* - *Every day* - *Several times a week* - *Once a week* - *Several times a month* - *Seldom/never* - *Rather not answer* |
| **How often do you eat any of the following foods, even in small quantities…**  **Fresh fruit, fruit and vegetable juice?**   - *4 or more times a day* - *2-3 times a day* - *Every day* - *Several times a week* - *Once a week* - *Several times a month* - *Seldom/never* - *Rather not answer* |
| **How often do you drink any of the following beverages, even in small quantities…**  **Tea or coffee with sugar, lemonade, cola, other soft drinks?**   - *4 or more times a day* - *2-3 times a day* - *Every day* - *Several times a week* - *Once a week* - *Several times a month* - *Seldom/never* - *Rather not answer* |
| **How often do you have a drink containing alcohol?**   - *Never* - *Monthly or less* - *2 to 4 times per month* - *2 to 3 times per week* - *4+ times per week* - *Rather not answer* |
| **[If the patient drinks alcohol]**  **How many alcoholic drinks did you have on a typical day when you were drinking in the last 12 months?**   - *0 drinks* - *1 to 2 drinks* - *3 to 4 drinks* - *5 to 6 drinks* - *7 to 9 drinks* - *10 or more drinks* - *Rather not answer* |
| **[If the patient drinks alcohol]**  **How often did you have six or more alcoholic drinks on one occasion in the last 12 months?**   - *Never* - *Less than monthly* - *Monthly* - *Weekly* - *Daily or almost daily* - *Rather not answer* |
| **Do you consume tobacco products?**   - *Yes, I smoke cigarettes* - *Yes, I smoke cigars* - *Yes, I chew tobacco* - *Yes, I use snus/snuff* - *I used to consume tobacco, but don’t anymore* - *No, I do not consume tobacco* - *Rather not answer* |
| **[If the patient consumes tobacco products]**  **How often?**   - *Less than 1 time per day* - *Between 1 and 5 times per day* - *Between 5 and 20 times per day* - *More than 20 times per day* - *Don’t know/not sure* - *Rather not answer* |
| **How would you rate your general health?**   - *Very poor* - *Poor* - *Fair* - *Good* - *Very good* - *Rather not answer* |
| **How would you rate your oral health?**   - *Very poor* - *Poor* - *Fair* - *Good* - *Very good* - *Rather not answer* |
| **Do you suffer from sensitive teeth?**   - *Often* - *Occasionally* - *Rarely* - *Never* - *Don’t know/not sure* - *Rather not answer* |
| **[If the patient suffers from sensitive teeth]**  **When does this sensitivity occur?**   - *While brushing teeth* - *Due to cold drinks, air or food* - *Due to hot drinks, air or food* - *When touching* - *Due to sweet foods* - *Other* - *Rather not answer* |
| **Have you experienced discomfort related to your mouth, teeth or dentures during the past 12 months?^**   - *Yes* - *No* - *Rather not answer* |
| **Have you experienced pain related to your mouth, teeth or dentures during the past 12 months?^**   - *Yes* - *No* - *Rather not answer* |
| **Do you spit blood or see blood when you brush your teeth?**   - *Yes* - *No* - *Rather not answer* |
| **Have you had difficulty eating food, including difficulty chewing or biting, due to problems with your mouth, teeth or dentures during the past 12 months?^**   - *Yes* - *No* - *Rather not answer* |
| **Have you had difficulty speaking or trouble pronouncing words related to your mouth, teeth or dentures during the past 12 months?^**   - *Yes* - *No* - *Rather not answer* |
| **Have you been embarrassed to smile or laugh due to problems with your mouth, teeth or dentures during the past 12 months?^**   - *Yes* - *No* - *Rather not answer* |
| **Have you had problems sleeping related to your mouth, teeth or dentures during the past 12 months?^**   - *Yes* - *No* - *Rather not answer* |
| **Have you limited participation in social activities, or had any difficulty enjoying the contact of other people, due to problems with your mouth, teeth or dentures during the past 12 months?^**   - *Yes* - *No* - *Rather not answer* |
| **Have you experienced difficulty carrying out your major work or role due to problems with your mouth, teeth or dentures during the past 12 months?^**   - *Yes* - *No* - *Rather not answer* |
| **If you work, have you taken time off because of problems related to your mouth, teeth or dentures in the past 12 months? This does not include time off taken for routine dental check-ups.**   - *Yes* - *No* - *Rather not answer* |
| **[If patient has taken time off work]**  **Approximately how many days have you taken off in the last 12 months?**  *Continuous* |
| **Have you ever felt that the appearance of your mouth, teeth or dentures affected your ability to interview for a job?**   - *Yes* - *No* - *Not applicable/never had a job interview* - *Rather not answer* |
| **Have you found that life in general was less satisfying due to problems with your mouth, teeth or dentures during the past 12 months?**   - *Yes* - *No* - *Rather not answer* |
| **My oral health has a good impact on my general wellbeing**   - *Strongly agree* - *Agree* - *Disagree* - *Strongly Disagree* - *Rather not answer* |

| **Dentist Questionnaire** |
| --- |
| **Does the patient visit your practice on a regular basis (at least once per year)?**   - *Yes* - *No* - *New patient/not applicable* |
| **What is the purpose of this visit?**   - *Check-up* - *Routine treatment* - *Emergency treatment* - *Other* |
| **How many teeth does the patient have present in his/her mouth?**  *Continuous* |
| **How many teeth with caries does the patient have?**  *Continuous* |
| **How many filled teeth does the patient have?**  *Continuous* |
| **How many missing teeth (out of 32 permanent teeth) does the patient have?**  *Continuous* |
| **Does the patient have any sealants present?**   - *Yes* - *No* |
| **How would you describe the patient’s periodontal status?**   - *Healthy* - *Gingivitis only* - *Shallow pocket (4-5mm)* - *Deep pocket (>6mm)* - *Mobile teeth* |
| **Does the patient have any other oral diseases or conditions (pick all that apply)?**   - *Craniofacial development abnormalities (e.g. cleft lip and/or palate)* - *Oral cancer/pre-cancer* - *Oral infections* - *Malocclusion* |
| **Does the patient have acid erosion?**   - *Yes* - *No* |

| **Dental Practice Questionnaire** |
| --- |
| **What is your age?**  *Continuous* |
| **What is your sex?**   - *Female* - *Male* - *[Locally appropriate options as determined by NDA]* |
| **What kind of practice do you work in?**   - *Private dental clinic* - *Public dental clinic* - *Public/private mixed clinic* - *Health centre that offers dental and other services* - *Hospital* - *Other* |
| **How many dentists work in this practice, including yourself? (If there are part time staff, please indicate the full-time equivalent i.e. two 50% workers = 1)**  *Continuous* |
| **How many other dental professionals, such as hygienists, nurses and surgery assistants, work in this practice? (please indicate the full-time equivalent for part-time staff)**  *Continuous* |
| **During an average day, how many patient contact hours do the dentists in your practice have?**  *Continuous* |
| **During an average day, how many patients visit your practice overall?**  *Continuous* |
| **For an average day, approximately what percentage of time do you spend providing check-up care?**  *Continuous* |
| **For an average day, approximately what percentage of your time do you spend in preventive practice (such as advice on diet, flouride use, oral hygiene or avoidance of risk factors such as tobacco and alcohol)?**  *Continuous* |
| **For an average day, approximately what percentage of time do you spend providing curative care?**  *Continuous* |
| **For an average day, approximately what percentage of time do you spend on administrative work?**  *Continuous* |
| **Over the course of an average year, how many hours do you spend on continuing education (articles, literature, internet search, courses, congresses)?**  *Continuous* |
| **How do you provide advice on cessation to those of your patients who use tobacco (pick all that apply)?**   - *Direct patient advice* - *Referral to tobacco cessation services* - *Do not provide advice* - *Don’t know/not sure* |
| **Which types of sealants do you use in your practice?**   - *Glass ionomer* - *Resin* - *Other* - *None* |
| **Do you know about the provisions on dental amalgam in the UN Minamata Convention on Mercury?**   - *Yes* - *No* |
| **Do you run an amalgam free practice?**   - *Yes* - *No* - *Don’t know/not sure* - *Rather not say* |
| **Do you have an amalgam separator?**   - *Yes* - *No* - *Don’t know/not sure* - *Rather not say* |
| **Which of the following restorative materials do you use in your practice (pick all that apply)?**   - *Amalgam* - *Composite resin* - *Glass ionomer cement* - *Ceramics* - *Gold based or other alloys* - *Other* |
| **Have you ever faced problems with the supply of any of the following restorative materials (pick all that apply)?**   - *Composite resin* - *Glass ionomer cement* - *Ceramics* - *Gold based or other alloys* - *Other* - *Never experienced supply problems* - *Don’t know/not sure* |
| **[If dentist has experience supply problems]**  **What issues have you faced?**   - *Lack of availability/supply* - *Cost too high* - *Poor quality materials* - *Poor supply chain management (condition and transport control)* |
| **How satisfied are you with being a dentist?**   - *Very satisfied* - *Satisfied* - *Neither satisfied nor dissatisfied* - *Dissatisfied* - *Very dissatisfied* |
| **How satisfied are you with your income?**   - *Very satisfied* - *Satisfied* - *Neither satisfied nor dissatisfied* - *Dissatisfied* - *Very dissatisfied* |
| **How satisfied are you with the education you received in university [adapt to local terminology, e.g. dental school]?**   - *Very satisfied* - *Satisfied* - *Neither satisfied nor dissatisfied* - *Dissatisfied* - *Very dissatisfied* |
| **How would you describe the importance of the collaboration in a "dental team" (dental assistant, dental nurse, dental hygienist, …)?**   - *Very important* - *Important* - *Neither important nor unimportant* - *Not important* - *Not important at all* |
|  |

Appendix 3 Dentist Information Kit


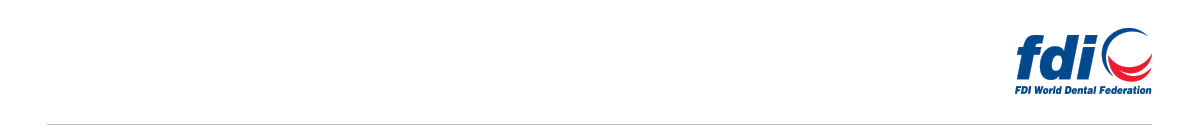


Contents

1. Introduction to OHO
2. Preparation steps:
   - Registration
   - Accessing the app
   - Selecting the patients
3. Completing the questionnaires


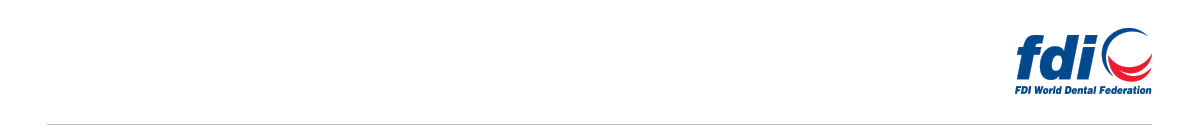


1.
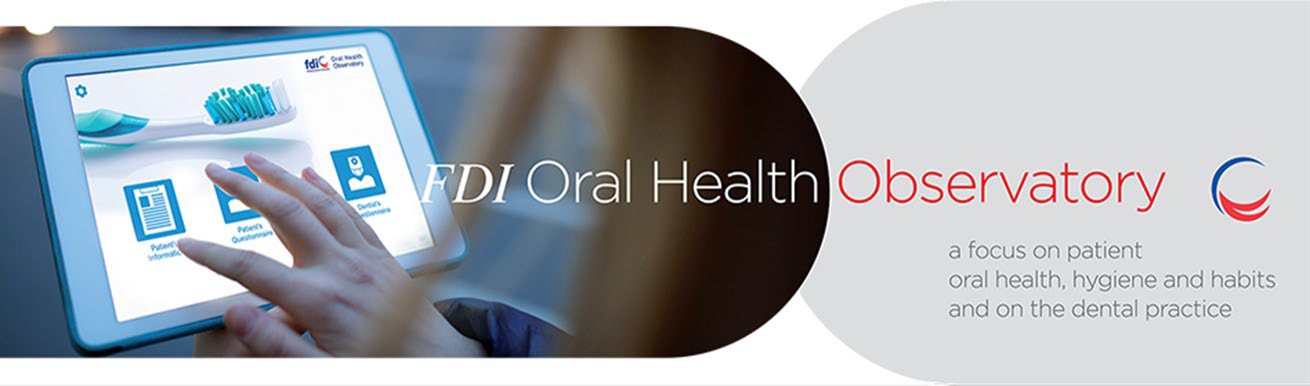
Introduction to OHO


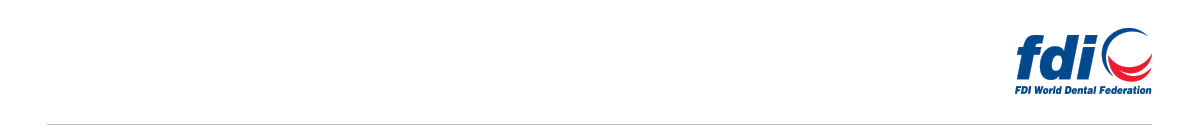


Objectives of the project

1. To collect data on:
   - Oral health status (children and adults)
   - Oral health and quality of life
   - Dental care provision
2. Use data to:
   - Support advocacy and inform national policy for improved dental care and services
   - Reinforce communication and awareness activities for oral health


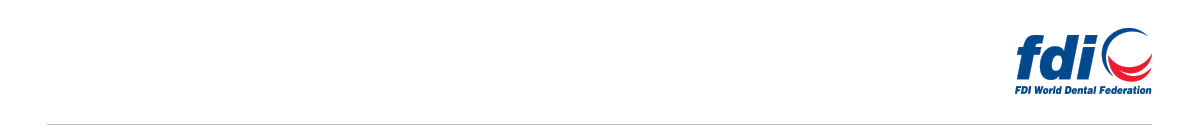


How OHO Works

1. You register your dental practice online and fill in a form with general information about the practice (one-time only)
2. You and the selected patients complete two separate questionnaires on the OHO app about oral health, behaviours and quality of life
3. You receive regular updates from FDI about the results from your practice and at the national level


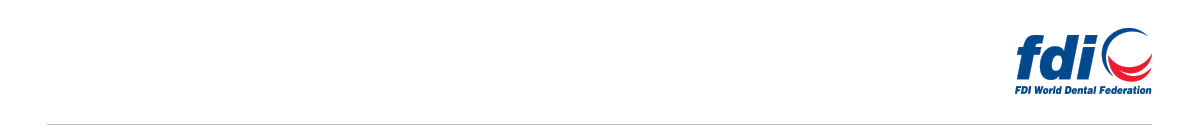


Three sets of questions

1. Dental practice questions (online – completed once)
2. Patient questionnaire (OHO app)
3. Dentist/dental assistant questionnaire (OHO app)


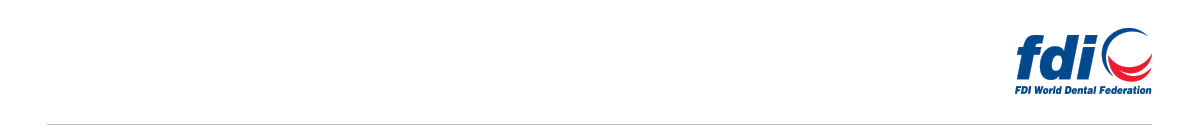


How the data will be used

- - All patient data is anonymous. Patients will not be identifiable from the information collected.
  - Data will be used by FDI and its project partners for advocacy and communication to improve oral health promotion and care.
  - You will receive updates with information about the patients surveyed in your practice, and overall data for your country


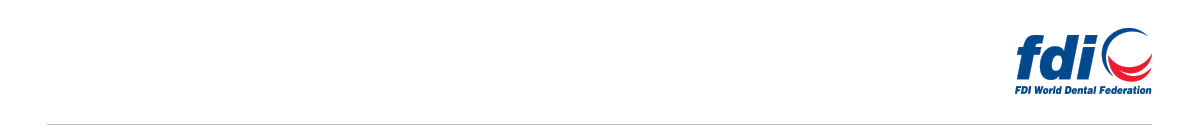


1.
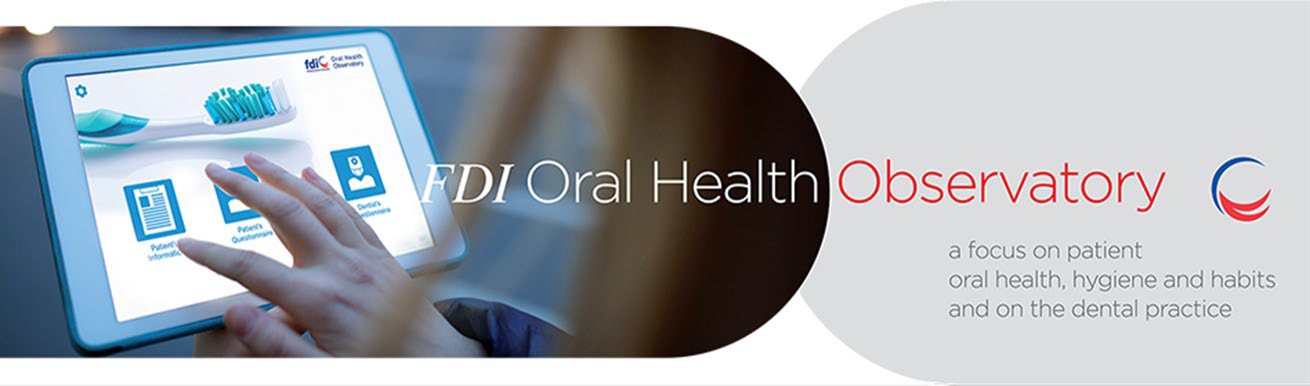
Preparation steps


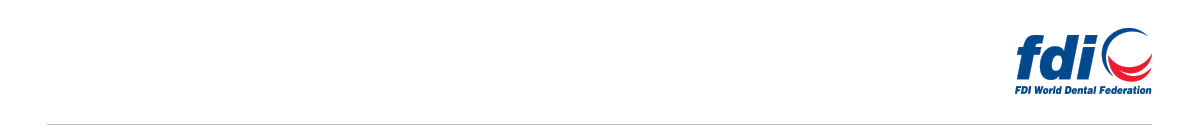


# Registration

Preparation steps


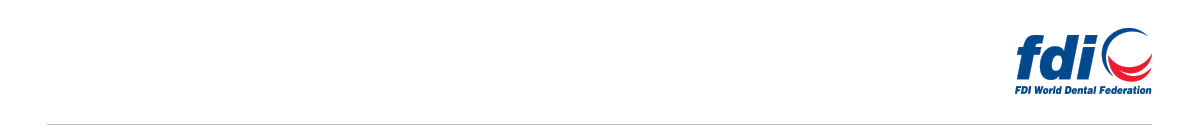


Registering for OHO

- - - You can register for OHO at <https://www.surveymonkey.com/r/OHO_practice_qs>
    - To register, you will need the username that FDI has sent to you by email
    - You will also be asked to respond to the dental practice questionnaire, which asks some general questions about your practice and will take around 5 minutes


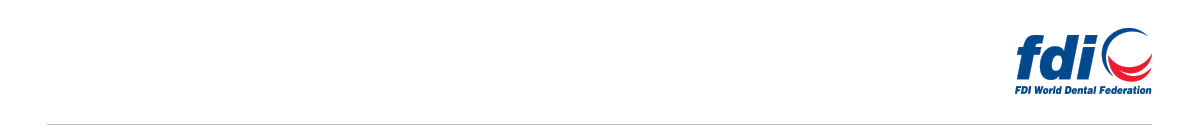


# Accessing the App

Preparation steps


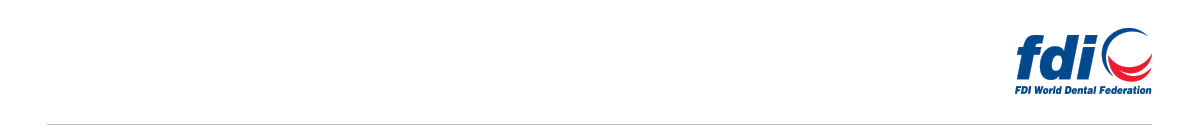


How to access the App

- - - Download and install the app by searching for ‘Oral Health Observatory’ or following the links below:
      - [App Store](https://itunes.apple.com/gb/app/oral-health-observatory/id1243418262?mt=8)
      - [Google Play Store](https://play.google.com/store/apps/details?id=com.ionicframework.oralhealthobservatory&hl=en)


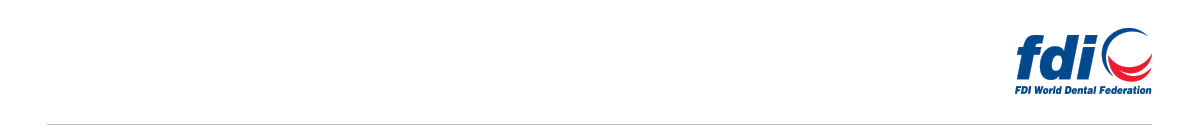


Logging in


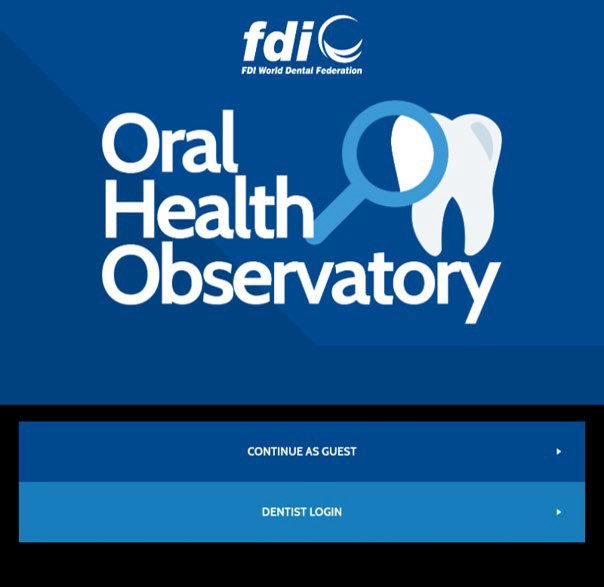

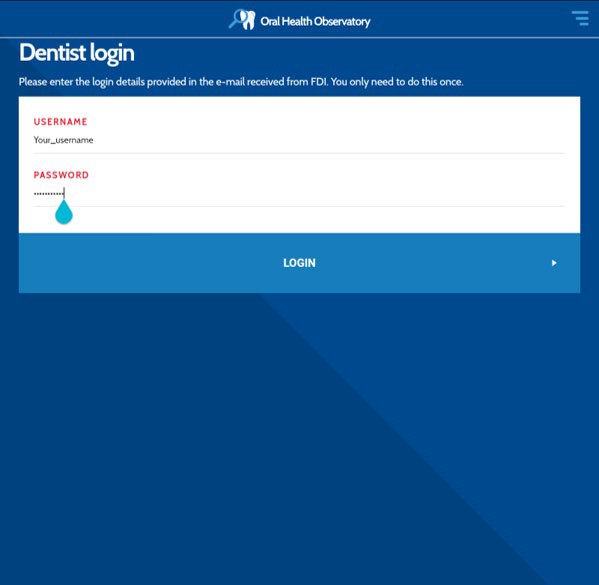


1. Open the OHO App and select “dentist login” on the welcome page
2. Enter the username provided by FDI and your password on the dentist login page


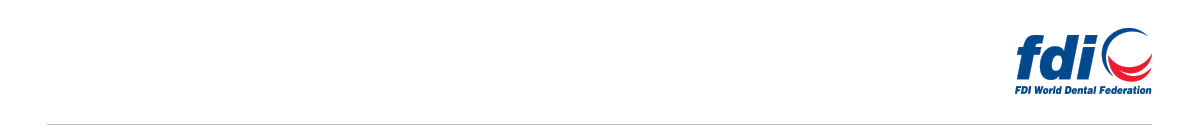


Changing language


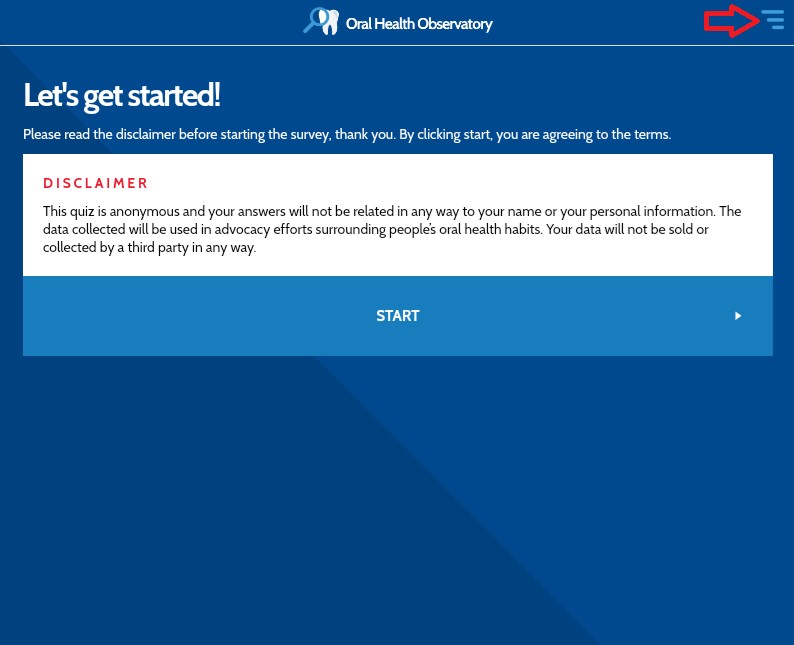


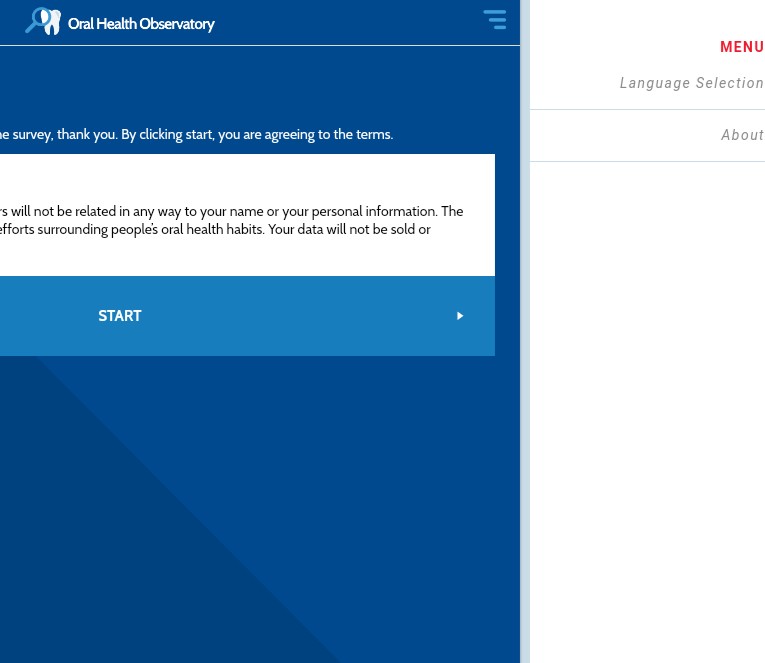


- - The app is available in [INSERT LANGUAGES] in [INSERT COUNTRY]. You can change the language according to your patient’s need/preference
  - To change language, press the menu bars at the top-right of the screen(see arrow) and then press ‘Language selection’


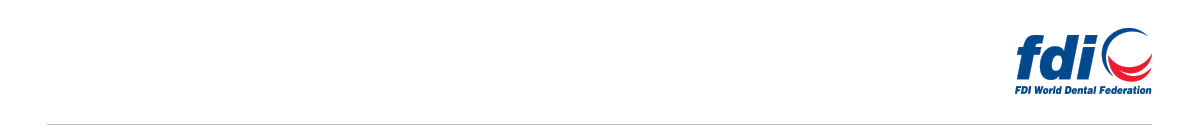


# Selecting the patients

Preparation steps


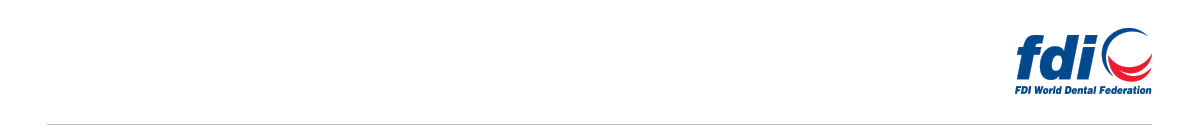


Selecting patients to participate

- - - In total, you should survey 50 patients during the course of the project.
    - You should survey one patient a day until the total of 50 is reached.
    - Patients should be selected at random. To achieve this, you should select patients according to the daily order, i.e.:
- Day 1 of study, first patient of the day is surveyed
- Day 2 of study, second patient of the day is surveyed
- …
- When the last patient of the day is reached, return to the first patient of the day


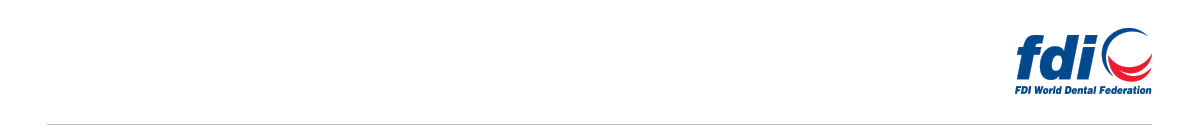


Refusals to participate

- - - Some patients may refuse to participate in the study. In this case, you should ask the following patient that day.
    - **Please be sure to record the number of patients who refuse to complete the survey and if possible their reason for refusal**. You should provide this data to the contact person at your national dental associations following completion of the project. This information will help when reporting the project results.


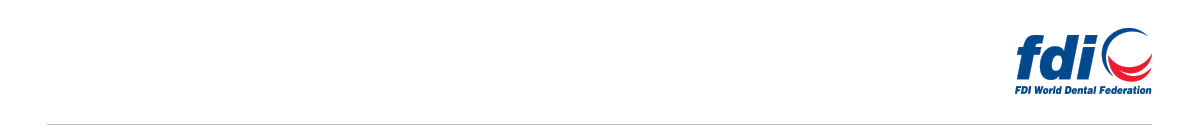


1.
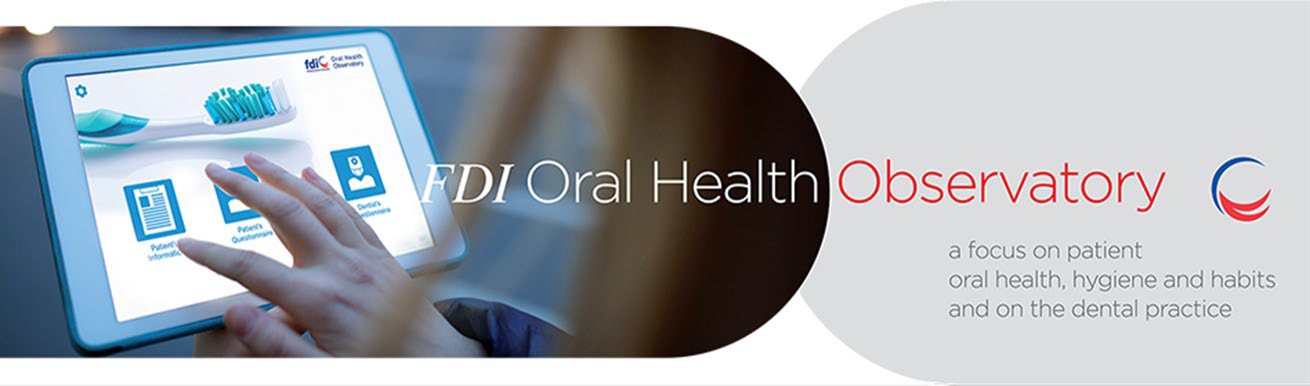
Completing the questionnaires


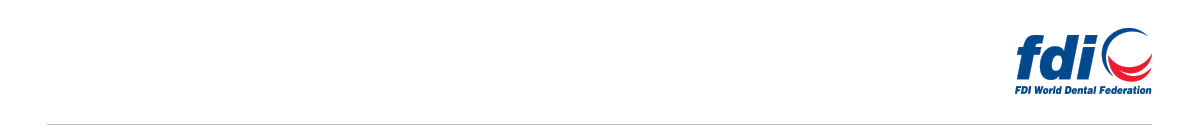


# Overal l work flow

Questionnaires


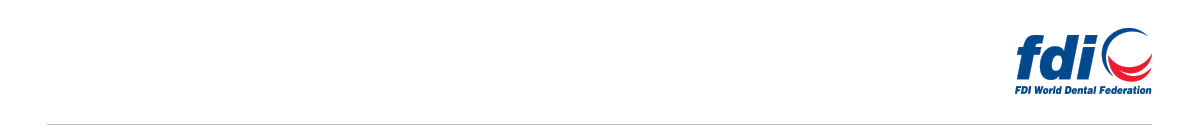


Workflow for patient and dentist questionnaire completion


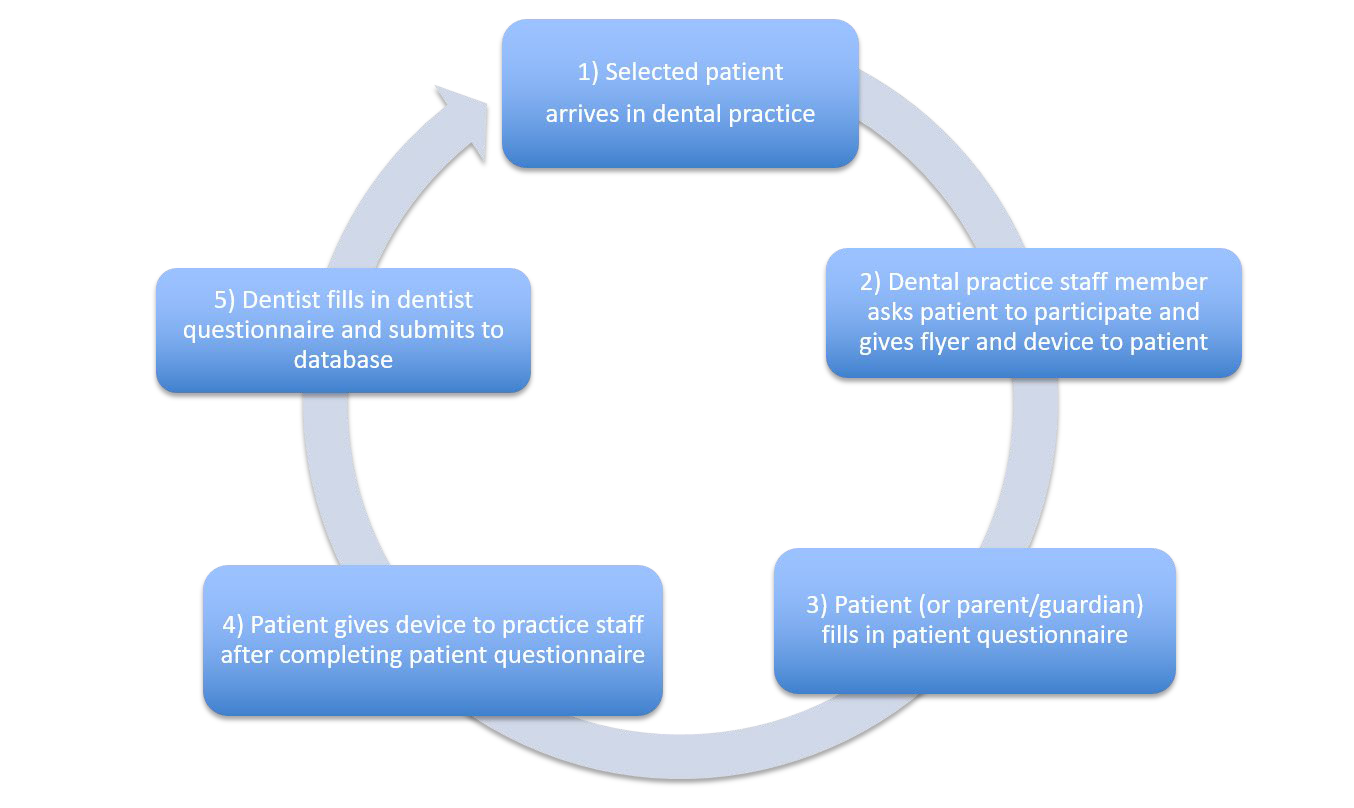


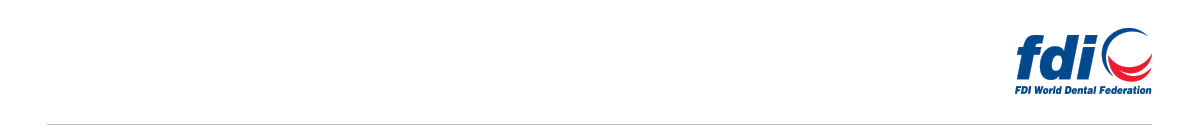


For each patient…

- - - Make sure the previous questionnaire has been completed and you have returned to the app start page
    - Give the project flyer to the patient so they are informed of the project and can complete the consent form (the consent form appears on the app before the questionnaire).


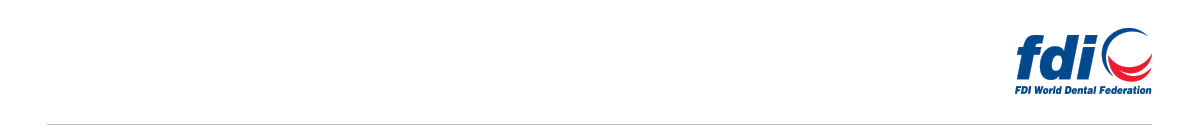


For patients under 12 years old…

- - - Their parent or guardian must complete the consent form and survey on their behalf.
    - Tell the parent/guardian they should answer the first question (“What is your age?”) with the age of the child.
    - The App will then automatically proceed with questions adapted for children.


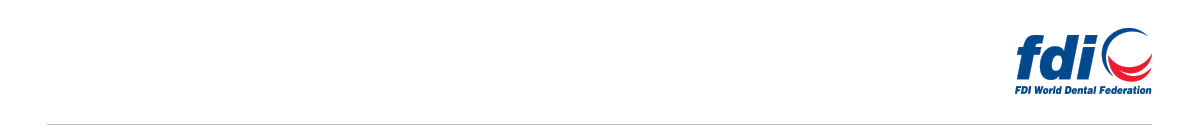


# Patient questionnaire

Questionnaires


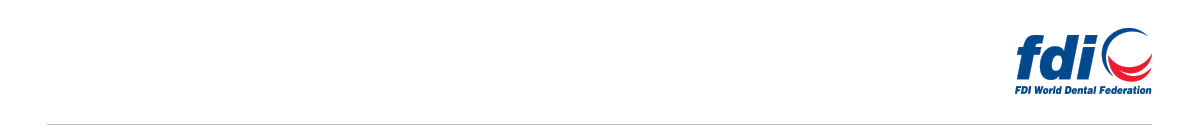


Patient questionnaire

- - - Completed by each patient using the OHO App, **before** their consultation or treatment.
    - Between 29 and 55 short questions (depending on responses and age) about:
- Demographic characteristics
- Dental visits
- Oral health behaviours
- Functioning and quality of life
  - - Estimated time of completion = 5-7 mins. Can be completed while waiting or at the beginning of the consultation


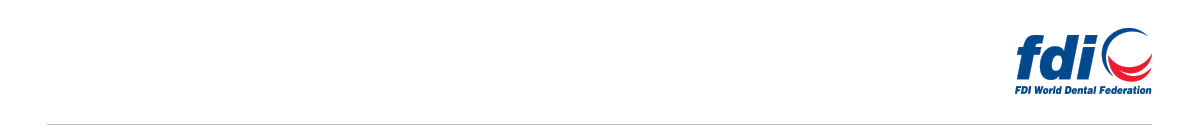


Taking the questionnaire: Start page


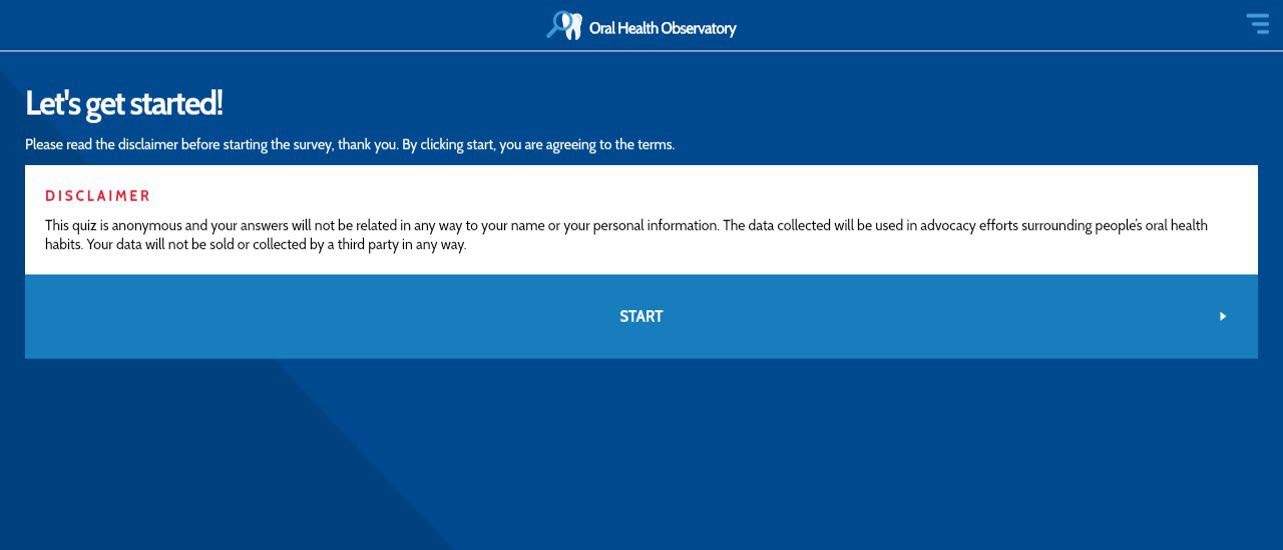


- - - Once you have logged-in, patients begin the patient questionnaire by selecting “start”
    - Please ensure the app is returned to the start page after each patient has completed the questionnaire


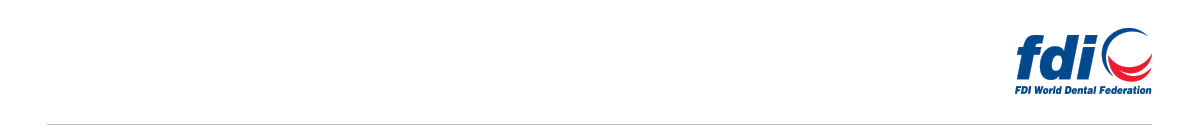


After completion: Proceeding to the dentist questionnaire


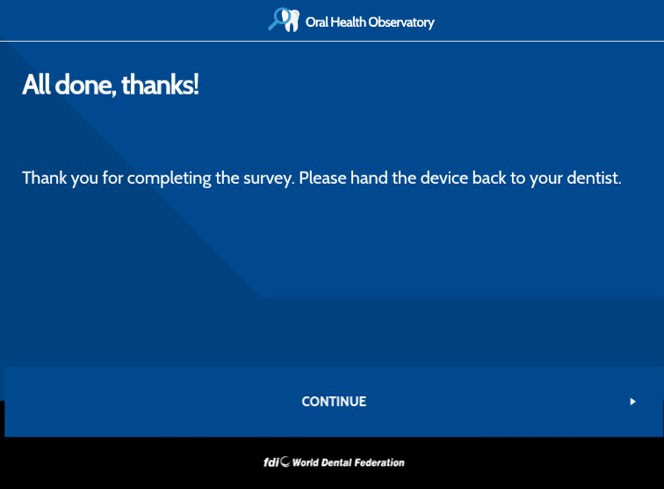

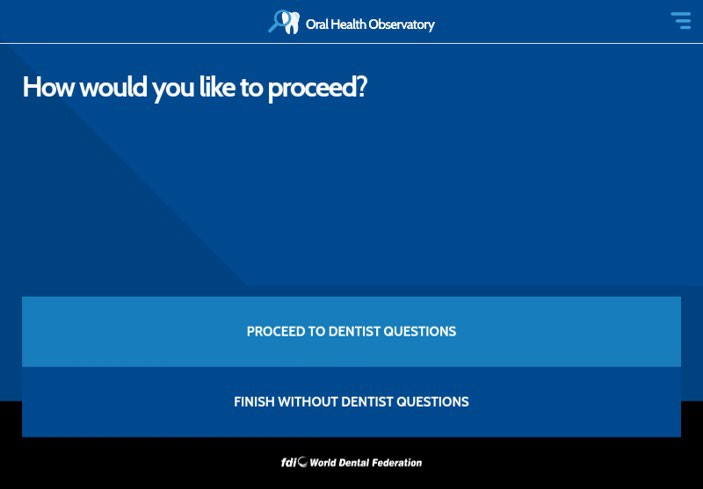


1. Once the patient has completed the patient questionnaire, they will be asked to hand the tablet or phone back to the dentist or a member of practice staff.
2. You should then select “proceed to dentist questions” to continue


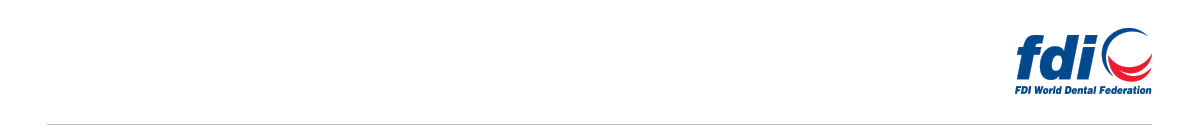


# Dentist/dental assistant questionnaire

Questionnaires


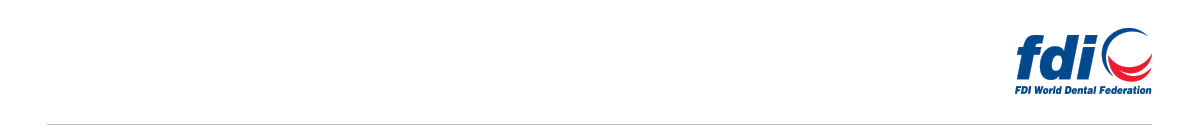


Dentist/dental assistant questionnaire

- - - Completed by the dentist or dental assistant for each patient, after the patient has completed the patient questionnaire
    - 10 questions about patient’s general information and clinical status
    - Estimated time of completion = 1-2 mins

Dentist Q4) Recording teeth with caries


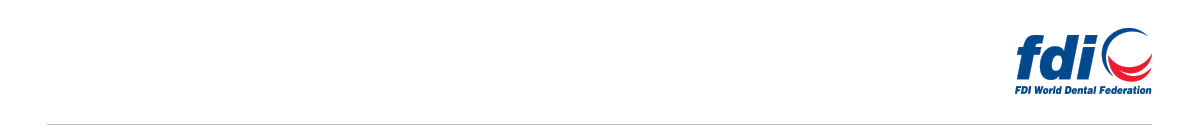

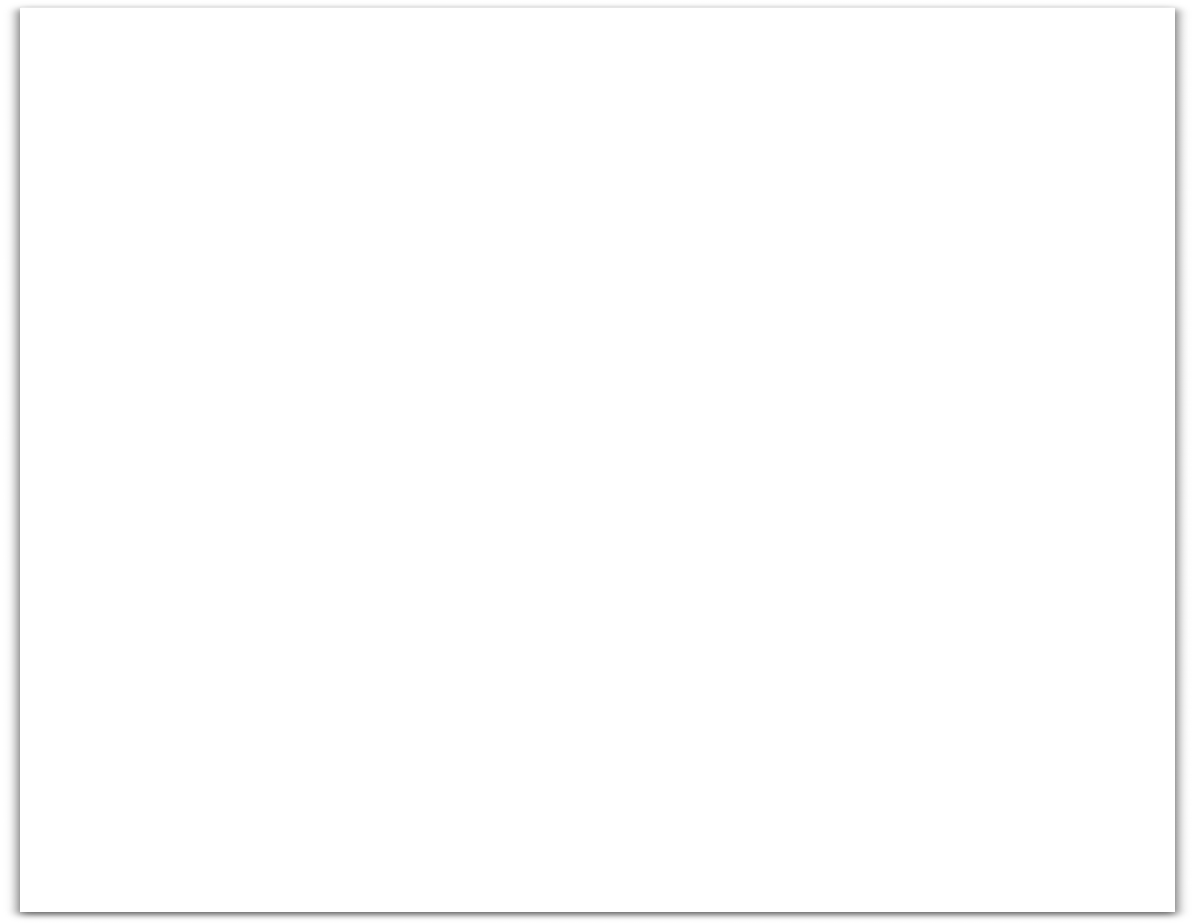

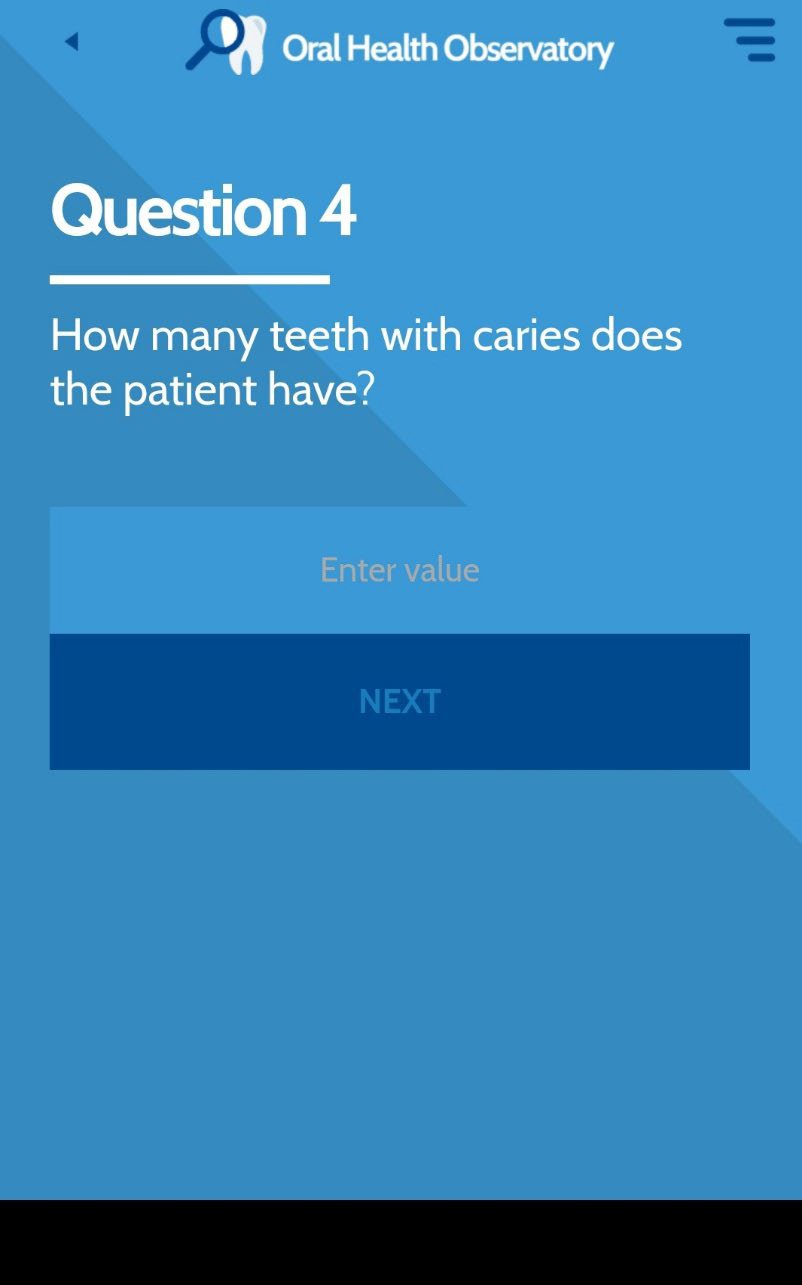


- The total number of teeth with caries should be recorded
- A tooth should be counted as having caries if it has:
  - A lesion in a pit or fissure, or on a smooth tooth surface, has an unmistakable cavity, undermined enamel, or a detectably softened floor or wall. OR;
  - A temporary filling, or a filling which is sealed but also decayed. OR;
  - A root lesion that feels soft or leathery upon probing.
- In case of doubt, do not record the tooth as having caries.
- [For more guidance on recording caries, please see the WHO Oral Health Surveys: Basic Methods Fifth Edition and p.44-4](https://apps.who.int/iris/bitstream/handle/10665/97035/9789241548649_eng.pdf?sequence=1)7 and p.95-99

## Dentist Q5) Recording filled teeth


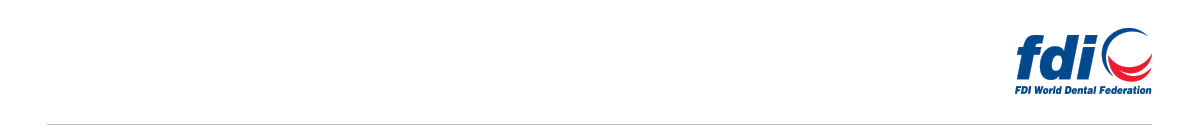

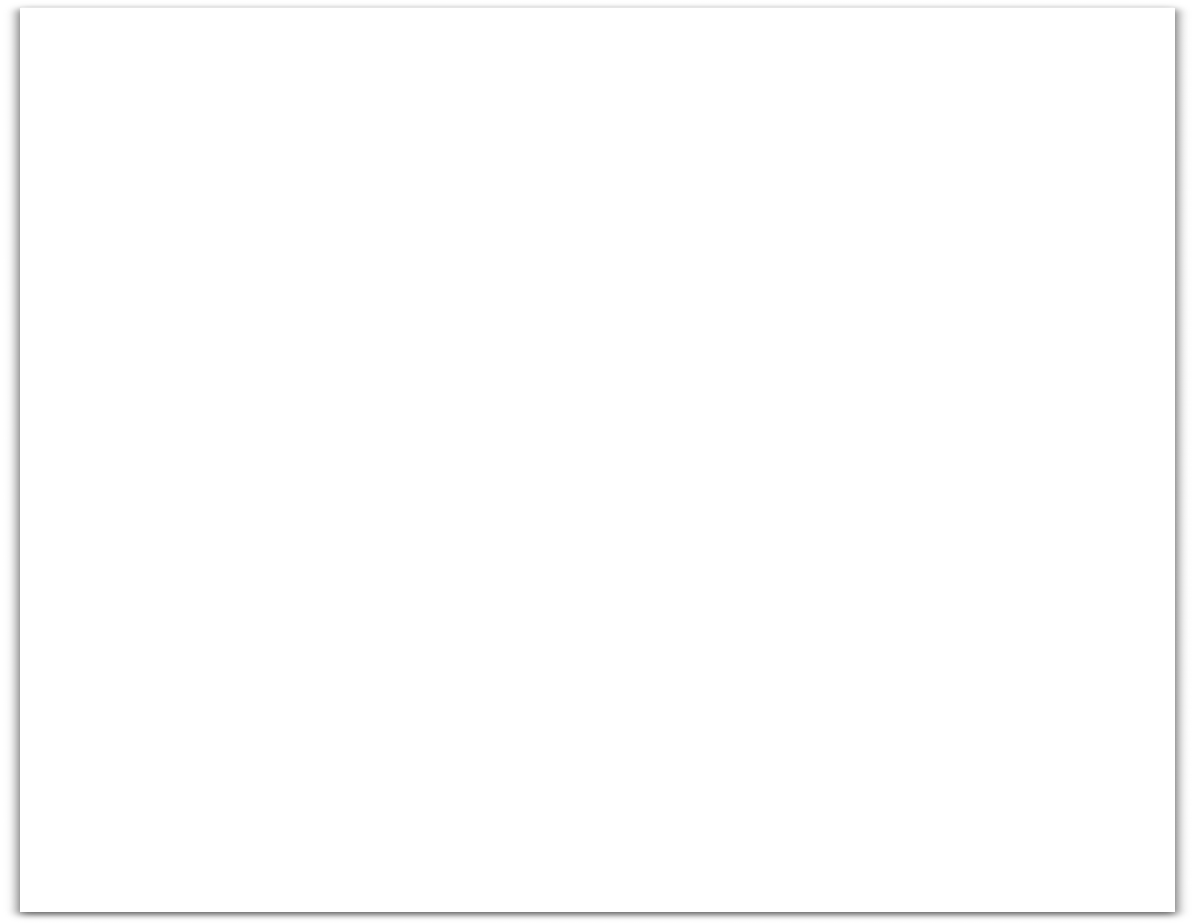

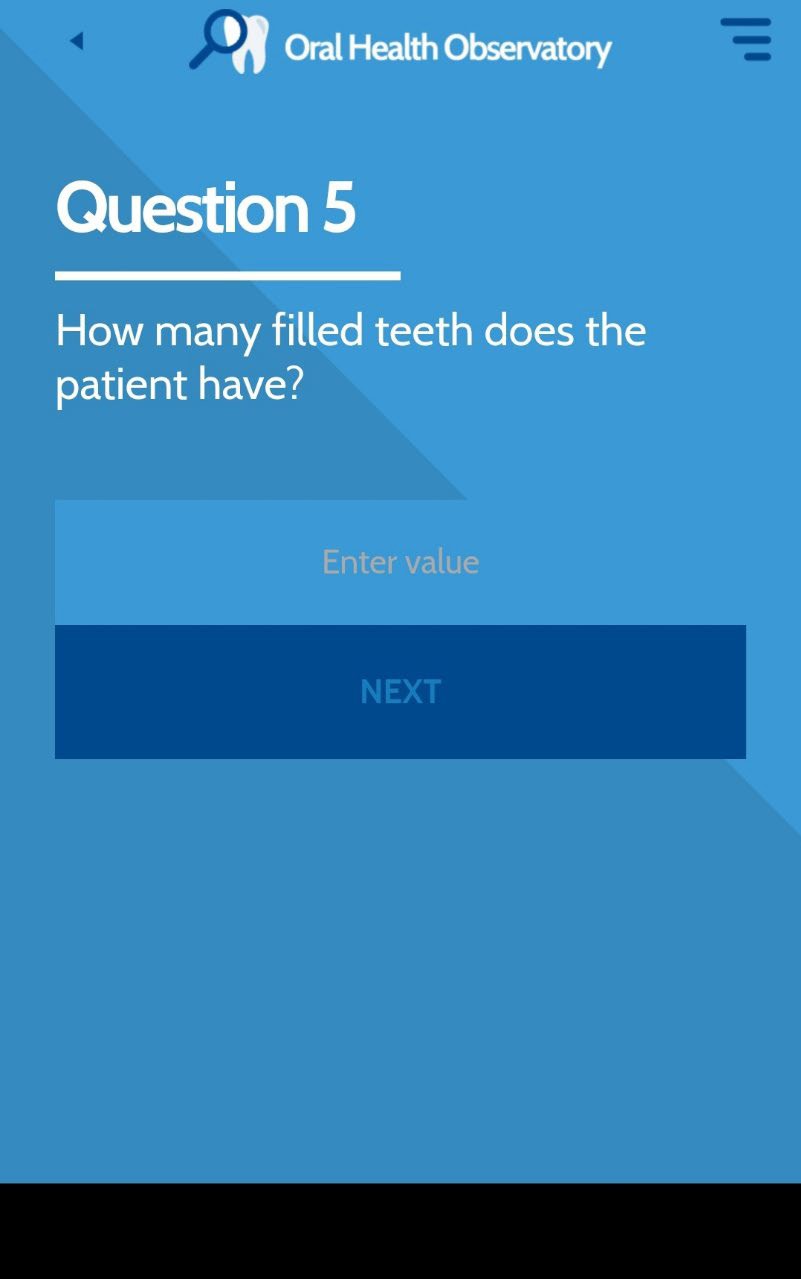


- - - The total number of teeth filled due to caries should be recorded.
    - A tooth should be counted as being filled if:
- “One or more permanent restorations to treat caries are present on the crown or root” AND;
- There is no caries present elsewhere on the crown or root.
- Any tooth restored for a reason other than caries should not be counted as filled.
- For more guidance on recording filled teeth, please see [the WHO Oral Health Surveys: Basic Methods Fifth Edition and p.45-47 and p.95-99](https://apps.who.int/iris/bitstream/handle/10665/97035/9789241548649_eng.pdf?sequence=1)

## Dentist Q6) Recording missing teeth


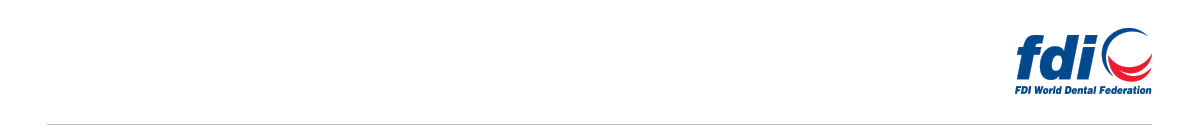

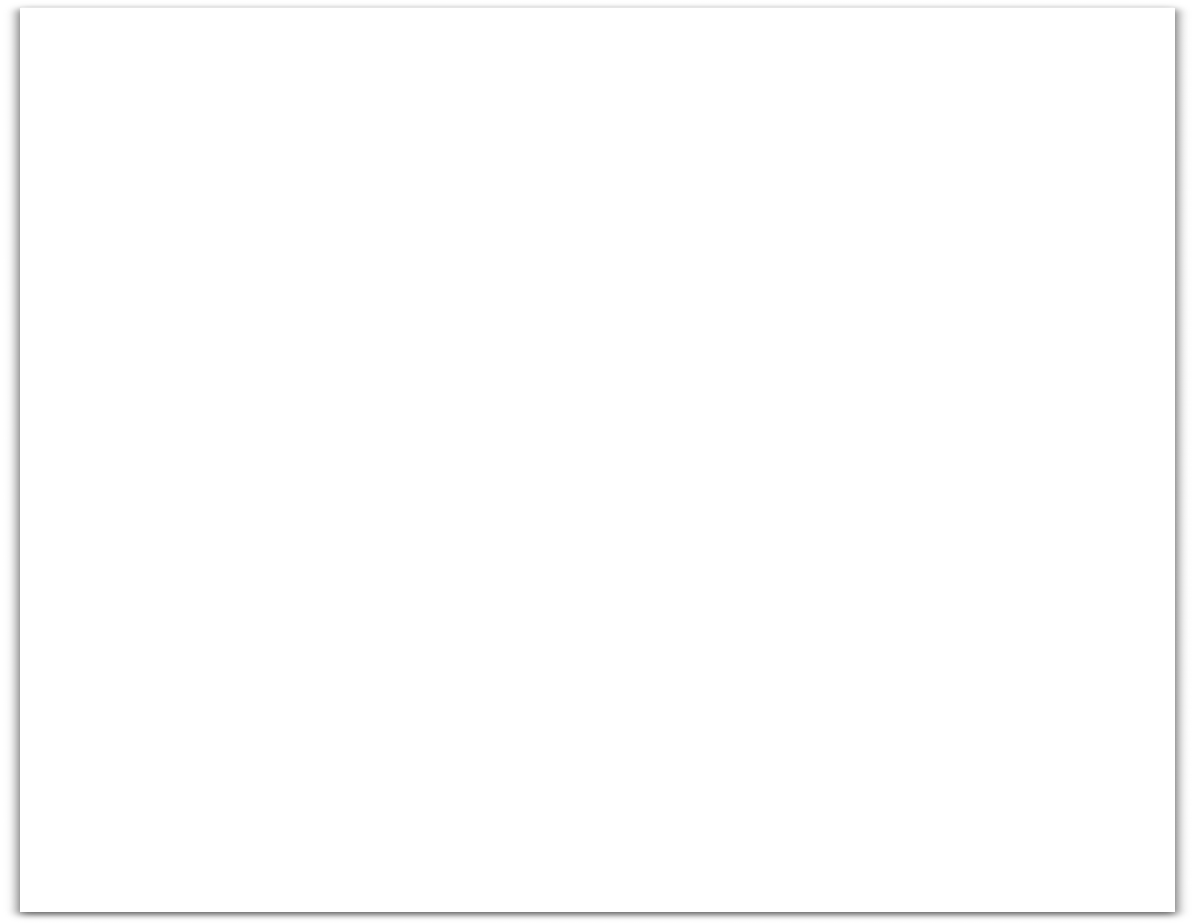

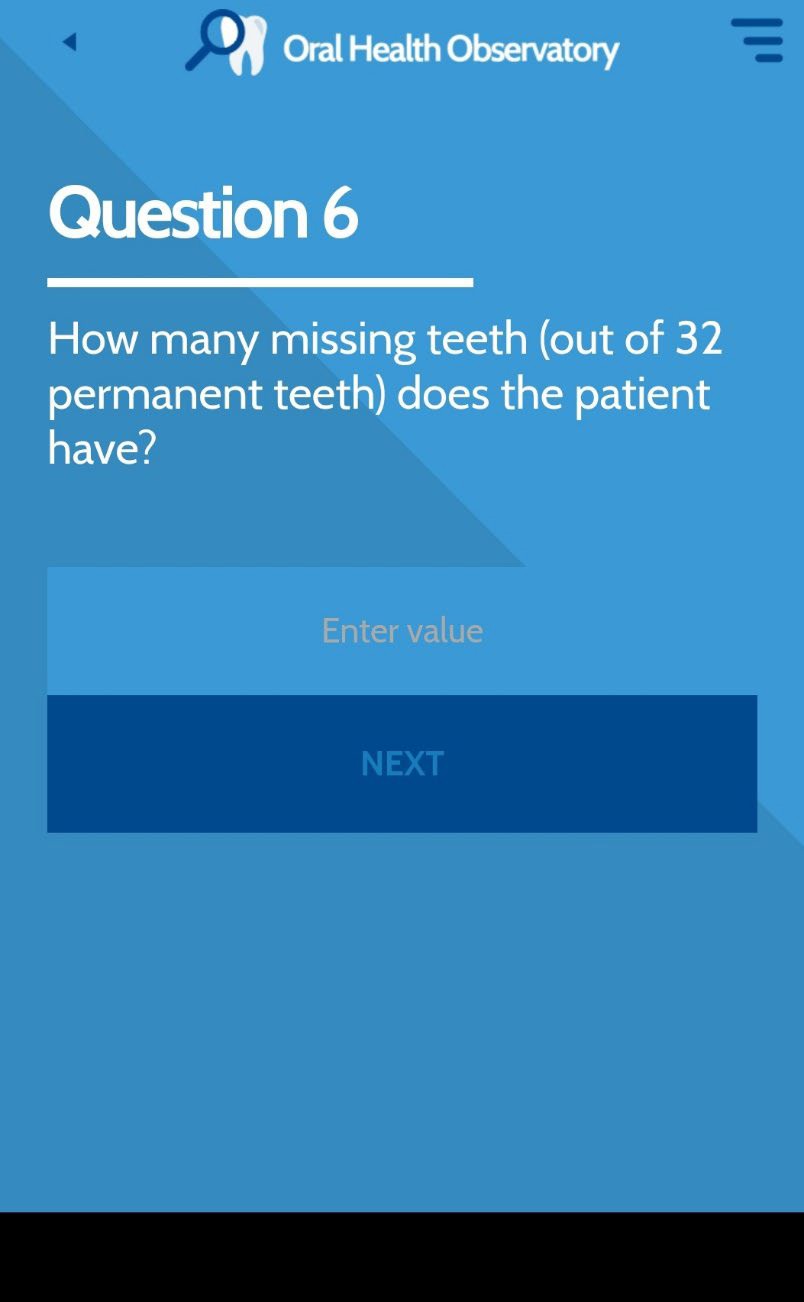


- The total number of missing teeth should be recorded
- A tooth should be counted as missing if:
  - In a patient **under** 30 years old, the tooth has been extracted due to caries.
  - In a patient **over** 30 years old, the tooth has been extracted due to caries, or is missing for any other reason (e.g. periodontal disease, orthodontic extraction, trauma…)
- In primary dentition, do not record teeth where normal exfoliation could be a reason for its absence.
- Knowledge of tooth eruption patterns, the alveolar ridge appearance and the caries status of surrounding teeth can help decide whether a tooth is unerupted or has been extracted.
- For more guidance on recording missing teeth, please see the [WHO Oral Health Surveys: Basic Methods Fifth Edition](https://apps.who.int/iris/bitstream/handle/10665/97035/9789241548649_eng.pdf?sequence=1) and p.45- 47 and p.95-99


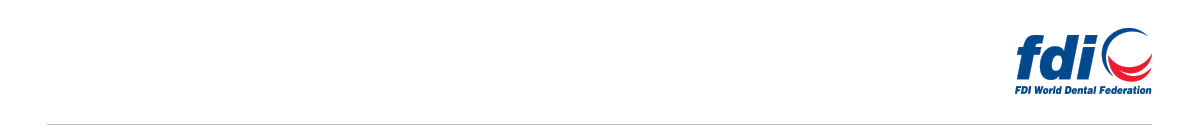


After completion: Submitting data and returning to the home screen


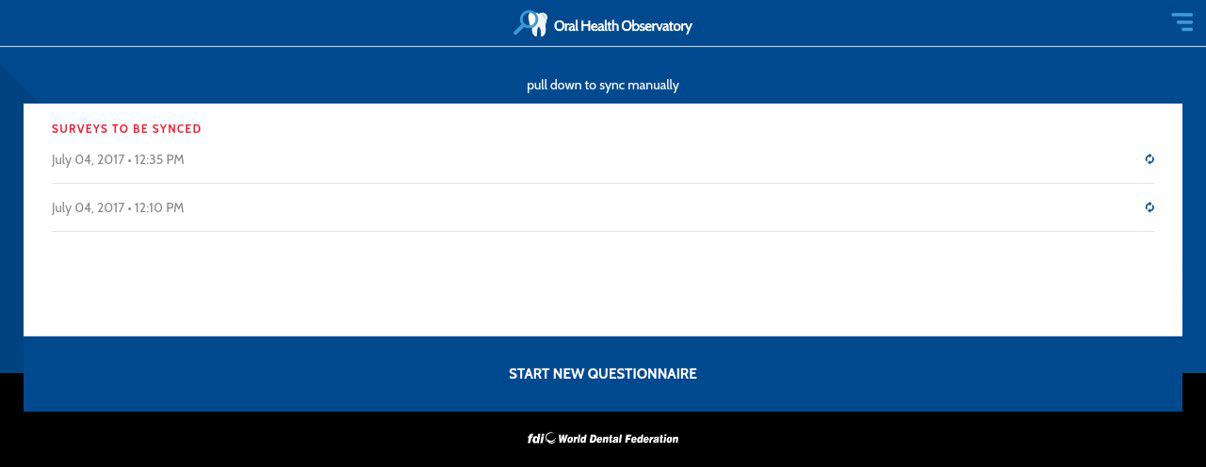


1. After you have completed the dentist/dental assistant questions, you can manually sync the surveys by pulling down on the sync screen. If you do not have a Wi-Fi connection, surveys will be stored and can be synced later on
2. You should then select “start new questionnaire”, which takes you back to the start page


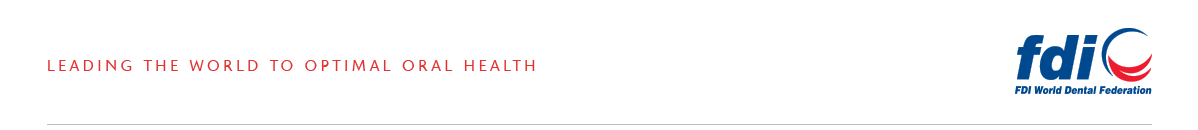


**Thank you for making**

**the Oral Health Observatory project possible!**
