# Supplementary material for: Using Standardised International Oral Health-Related Datasets in 6 Countries
Source: Int Dent J. 2024 Feb 2;74(3):647–55. doi: 10.1016/j.identj.2024.01.001 (PMC11123530; doi:10.1016/j.identj.2024.01.001)
Supplement: Supplementary file 3 [file mmc3.pdf]

Oral Health Observatory Project: Research Protocol

Summary

The Oral Health Observatory (OHO) project seeks to collect standardized data on oral health through mobile app based surveys in several countries. Data will be collected on dental patients' perceptions of their oral health and its impact on their quality of life, dental visiting habits, and oral health behaviours. Data will also be collected on the patients' oral health status, allowing for comparison of patients' perceptions with clinical measurements. At a global level, there is a lack of standardized, comparable data for these elements.

Data collection for OHO will be done via a network of dentists using a mobile application. The application will contain two questionnaires: one completed by the patient relating to their demographic information, subjective perceptions of how their oral health affects their quality of life, oral health behaviours, and dental visiting patterns; and a second completed by the dentist about the patient's oral health status. Two different methodologies for selecting participating dental practices will be used according to the country's capacity and needs.

The smaller, general study will select practices to represent the population distribution among administrative divisions, and the extended study will take a representative sample of all dental practices in the country. In both cases, patients visiting participating dental practices will be selected randomly, with any patient over 3 years old, resident of the study country, and able to give informed consent eligible to take part. A parent or guardian will complete the survey on behalf of children under 12 years old. All patient data will be collected and stored anonymously. Additional data relating to the dental practice will be collected via an online registration portal.

The data collection period will run for three months. Following this the project will be evaluated to assess the practicalities of administering the OHO questionnaire in the dental practice setting using a mobile app, which can inform subsequent data collection under the OHO project, and similar research projects in future. Results will also inform communication and advocacy work about oral health and patient perceptions at the national and global level.

1. Rationale and Background

The Oral Health Observatory (OHO) is an international project which seeks to collect standardized data on dental patients' perceptions of their oral health and its impact on their quality of life, dental visiting habits, and oral health behaviours in several countries worldwide. The project will also collect data about patients' oral health status, allowing comparison of perceived oral health status and clinical information. Currently, there is a lack of internationally comparable data for these elements of oral health and dental care. Using a common questionnaire, housed on a mobile app and administered in dental practices, OHO will collect data to understand and compare the situation within and between countries. This first phase of the OHO project will collect initial data and evaluate the feasibility of the study design. The project evaluation and results will be used to assess the implementation of an oral health survey in the dental office using a mobile app, and the relevance of the questions used in the current OHO questionnaire. These outcomes may inform the development of a subsequent and extended phase of the project. Results will additionally be used for advocacy work at the national and global level, with the aim of better understanding and mitigating oral disease and its impact on quality of life.

The study is led by the FDI World Dental Federation, and will be coordinated at the national level by several of FDI's member national dental associations (NDAs). Each NDA will be responsible for the implementation of the study within their country. Data will be collected in general dental practices that are selected by the NDA. The responsible person for the study within each dental practice will ensure that it is carried out according to the relevant sections of the study protocol, namely the patient selection methodology and ethical considerations.

OHO is funded by Glaxosmithkline services unlimited (GSK). Funds are managed by FDI and distributed to support the study implementation as required.

2. Study Design

OHO is a cross-sectional, analytical observational study. The study population is patients attending dental clinics in several countries. Participants will be selected randomly among all patients attending the participating dental clinics during the study period (see 'Methodology' section). Inclusion criteria are that patients are aged over three years old, able to give informed consent, and resident in the study country. For young children who are unable to give informed consent, parents will give proxy consent, with the age limit for proxy consent adapted to national regulations as necessary. Data collection will take place until the required number of patients has been reached (see 'Methodology' section).

3. Methodology

Patient data will be collected in dental practices using a mobile application which contains the two core questionnaires, one to be completed by patients and one by dentists. A third questionnaire will collect basic information about the dental practices where the survey will be carried out. The full list of questions in each questionnaire is provided in Annex A. The questionnaires have been developed by the OHO Task Team (see 'Quality Assurance' section) to ensure collection of the most relevant information in the areas of interest, and to ensure alignment with FDI's new oral health definition. The OHO experts, and experts from FDI's Vision 2020 project who worked on the FDI oral health definition, agreed which core elements of oral health should be covered by the survey to best meet the study goals. Questions from several commonly used oral health surveys[2-7] were reviewed to inform their development. Participating NDAs also had the opportunity to review questions and propose changes and additions, considering the relevance and appropriateness of questions to the national context. Any additional questions that were proposed by NDAs for use in their country, or the project funder GSK, were reviewed by the project experts who decided whether they should be included.

Two different sampling methodologies have been developed to allow implementation in countries with different capacities for carrying out the study. The smaller sampling method, referred to as the 'General Study', selects dental practices to represent the different administrative divisions and urban and rural areas according to population distribution. It is the minimum standard required for countries to participate in the study. The larger sampling method, referred to as the 'Extended Study', selects a sample that is representative of all dental practices within the country. The two different methodologies are explained in greater detail below:

General Study: Sampling method

The General Study sampling methodology is based on the pathfinder method, described in WHO's Oral Health Basic Survey Methods Fifth Edition.[7] Pathfinder is a stratified cluster sampling method that covers the most important population subgroups who are likely to have different oral health statuses. It suggests selecting between 15 and 25 data collection sites per country. For the purposes of OHO general study, the methodology has been developed further so that sites will be selected according to population distribution among all administrative regions of study countries and, where possible, urban and rural areas.

To select the study sites, dental practices (DPs) will be clustered according to the primary administrative division (e.g. state, province) in which they are located. The number of DPs to be selected in each cluster will be set according to the proportion of the national population living in that administrative division. A total of between 20 and 30 DPs will be selected per country, with the precise number being calculated to minimise rounding errors and to ensure representation of the least populated primary divisions. NDAs will then randomly select the required number of DPs per cluster. If a DP doesn't agree to participate, the NDA continues the process until a DP accepts. If

no DP with the required letter agrees, FDI will generate a new random letter and communicate this to the concerned NDA.

Where the necessary statistics are available, dental practices within each division will also be selected to represent the proportion of the population living in different area types (urban, semi-urban, and rural). Within each cluster, practices will be organized into sub-clusters according to whether the secondary administrative division (e.g. municipality, district) in which they are located is mostly urban, semi-urban, or rural. The number of practices to be selected in each cluster, calculated as outlined above, will then be divided among the sub-clusters according to the proportion of the population living in urban, semi-urban, or rural areas. Random sampling will then be used to select the required number of dental practices per sub-cluster. The number of different sub-cluster types and name will vary according to available data and categorization used in official records.

NDAs will recruit dental practices and coordinate the study implementation. This will include distributing necessary materials and providing support for carrying out the project according to the study design.

In each dental practice, patients will be selected randomly according to the running order of patients (first day, first patient is asked to participate; second day, second patient is asked to participate, and so on). One patient will be surveyed per day until the total of 50 patients per practice is reached. If a patient declines to participate, the following patient on that day will be asked to participate until someone accepts.

Extended Study: Sampling method

The Extended Study will use a probabilistic approach to select a sample which is representative of the dentist population of the country. Dental practices are randomly selected among all dental practices within the NDA network. The minimum size of the sample of dental practices is described by *n* in the following formula:

(1)

$$n \geq \frac{(N)(p)(1-p)}{(N-1)(D)+p(1-p)}$$

Where:

$$D = \frac{(confidence\ interval)^2}{Z^2}$$

n = sample size

N = population of dentists, according to UN World Health Statistics, 2014[8]
p = 0.5 (this assumes patients are as likely to choose one answer as another)
Z = area under normal curve corresponding to the desired confidence level (CL).
Confidence interval (CI) = 1-CL

In most study countries, a 90% CI would require a sample of between 67 and 69 dental practices (DPs), and a 95% CI would require a sample of between 362 and 385 dental practices, depending on the country's population of dentists. Given the resources available for the project, a 90% CI will be used to ensure manageability. The required number of dental practices will be randomly selected from a list of all dental practices in the country accessible to the NDA concerned. A list of 67 to 69 letters will be randomly generated using Microsoft Excel and shared with the NDA concerned. Then, the NDA randomly selects one DP located on a street whose first letter corresponds to each of the letters. If the DP doesn't agree to participate, the NDA continues the process until a DP accepts. If no DP with the required letter agrees, FDI will generate a new random letter and communicate this to the concerned NDA.

The number of patients to be surveyed in each dental practice will be set according to the total number of patients that will visit the practice over the study period. Examples of the number of patients required for a 90% CI for different practice sizes is shown below. These number are calculated using formula (1) shown above.

In a dental practice with an estimated number of patients seen during the study period of:	Then the number of patients that need to be surveyed for a 90% CI is:		
50	29		
200	51		
500	60		

In each dental practice, patients will be selected randomly according to the running order of patients as in the General Study.

Both studies: In-practice procedure

In both the General and Extended Study, the randomly selected patients will be asked to participate in the study upon arrival at the dental practice. Firstly, selected patients will be asked by the dentist or a member of practice staff if they are willing to participate in the project, and provided with

relevant information in line with the consent procedure (see 'Safety Considerations, Ethics, Consent and Privacy' section). If they accept to participate, the patient will complete the first questionnaire, relating to their perception of the impact of oral health on their quality of life, oral health behaviours, dental visits, and basic demographic information, prior to their consultation with the dentist. Ideally this will be done during the waiting time, but in some instances this may not be possible. Participating dental practices will be requested to consider this in their planning to allow completion of the questionnaire before the consultation, with the aim of minimizing missing data. Upon completion, the patient will return the mobile device to the member of practice staff. The dentist will proceed with the treatment or check-up as planned. During the treatment or check-up, the dentist will then complete the dentist questionnaire using the mobile device, which will collect data about the patient's basic oral health status.

4. Safety Considerations, Ethics, Consent and Privacy

Study participants will receive an information sheet describing the purpose of the study, the information to be collected, and their right to withdraw at any time, prior to beginning the questionnaire. The text of this document is provided in Annex B. They will also complete an informed consent form on the mobile application prior to beginning the questionnaire, the text of which is provided in Annex C. The information sheet and consent form will be provided in relevant national languages in each study country.

5. Data Management and Statistical Analysis

Collected data will be transferred from the OHO mobile application to the FDI secretariat in Geneva, Switzerland. It will be stored on FDI's server. No identifiable patient data will be collected, and it will therefore be transferred and stored anonymously. Data collected from the online dental practice questionnaire will also be transferred to, and stored on, FDI's server. The dentist submitting this data will be identifiable, however no sensitive information will be collected by this questionnaire (see 'Dental Practice Questionnaire' in Annex A). NDAs will also be able to access the raw data, which FDI will transfer on request. Responses to 10 questions of interest will also be shared with the project funder GSK. All data will be used solely for research purposes, and not for commercial or marketing reasons.

Data will be treated for bivariate and multivariable analysis to assess the relationship between variables. Areas of analysis will be decided upon once data is collected, with possible areas including:

- The determining and moderating factors of perceived oral health impact on quality of life, looking at the influence and interactions of oral health status, dental visiting patterns, socioeconomic status (SES), and demographic variables.
- The determining and moderating factors of dental visiting patterns, looking specifically at the influence and interactions of SES, demographic variables, and patient's perceptions of oral and general health.
- The determining and moderating factors of oral health status, looking at the influence and interactions of dental visiting patterns, oral health behaviours, SES, and demographic variables.

6. Quality Assurance

The OHO questionnaires were developed through a consensus process with several international experts in the field. The experts are members of the OHO project Task Team, and members of the FDI Vision 2020 Think Tank who worked on the FDI new oral health definition. Members of the OHO Task Team who worked on the final questionnaire development are:

- Dr Michael Sereny, Chair FDI Dental Practice Committee
- Dr Georgios Tsakos, Reader in Epidemiology and Public Health, University College London
- Professor David Williams, Research Professor of Global Oral Health, Barts and the London School of Medicine and Dentistry, Vice-chair FDI Science Committee

To ensure legibility, any translation of project materials will be done by professional translators and reviewed by experts in the national dental association for both language and content. Questionnaires will be translated into relevant national languages in each study country.

All participating dental practices will receive a training manual to standardize the data collection procedure within the practice. This will include the procedure for explaining the study to patients, and for collecting the clinical data about the patient's oral health status. NDAs are responsible for providing support to dental practices as needed and verifying that the study has been correctly carried out within the practices.

7. Expected Outcomes

OHO will achieve three separate outcomes:

- **Assess the implementation of the survey and the relevance of the questions used in the current OHO questionnaire:** The project evaluation will allow assessment of the implementation of such a survey in the dental practice using a mobile app. This evaluation can help inform the study design for future phases of OHO, and possibly similar projects. The project evaluation and results from the questionnaire will also allow assessment of the

relevance and construction of the OHO questionnaire and modification for subsequent study phases.

- **Better understand the impact of patients' oral health status on their perceived psychosocial function, physiological function and overall quality of life:** Comparison of oral health measures recorded by the dentists and the functional and quality of life measures submitted by the patient will give a better understanding of the individual and societal burden of poor oral health.

- **Collect standardized data on oral health status, access to care, and oral health behaviours of patients in participating countries:** There is a lack of quality, standardized, and recent data looking at the prevalence of oral health conditions, access to dental care and oral health status in many countries. OHO will collect such data and allow comparison of these factors with each other and demographic variables. This will give an insight into how oral health and dental care vary with geographic location, socioeconomic status, and other factors, within and between countries.

8. Dissemination of Results

Results will be used to create a range of communication materials for use at the global and national levels. These materials will highlight findings about the impact of oral diseases on function and quality of life, and disparities in oral health, access to care, and behaviours among different population groups. They can be used to inform policies that address the determinants of poor oral health in populations in need, and encourage the prioritization of oral health based on the quality of life impact. An evaluation of the project implementation will be disseminated to provide interested parties with an assessment of the project design.

9. Project Timeline

The below project timeline displays the different aspects of the project, planned time of completion, and responsible party for completing the task (FDI staff, NDAs, and OHO Task Team). As this timeline applies to all participating countries who may begin or complete tasks at different times, some aspects overlap:

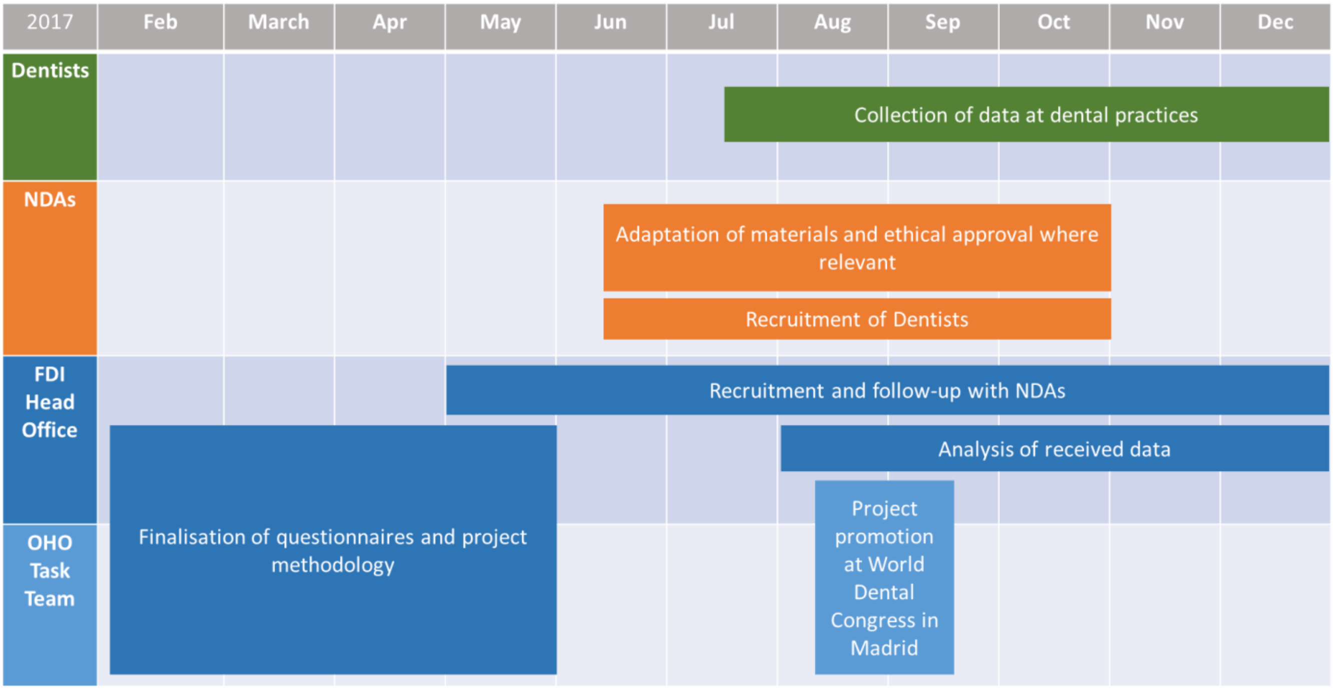

10. Budget

Below is the budget for implementation of OHO in [insert country name]:

[Insert NDA budget]

Annex A: OHO Questionnaires

The following are the core OHO Questionnaires. In some countries, some questions may be slightly modified, or some questions added to suit national context and needs.

[Insert final questionnaire]

Annex B: Participant Information Sheet

The following is the text of the participant information sheet, which will be given to all potential participants by the person responsible for the study in the dental practice:

What is the Oral Health Observatory (OHO)?

OHO is an international research project, mapping oral health and dental care across several countries worldwide. The project looks at people's oral health, how this impacts their daily life, and how it is related to their dental care and environment.

OHO is a project of the FDI World Dental Federation, and is run in [insert country] with the collaboration of [insert NDA name].

Why participate in OHO?

The information collected by OHO will be used to advocate for policies to improve oral health and dental care in [insert country]. By participating in the survey, you are helping provide an understanding of oral health in [insert country], and building a powerful message about how things could be improved.

How does it work?

Your dental practice will provide you with guidance to complete the survey. Using a tablet or smartphone, you will complete some basic information about yourself and how your mouth and teeth impact your daily life. In total, the survey will take **around 5 minutes** to complete. Your dentist will then complete a survey about your mouth and teeth condition. The answers you and your dentist give will be collected by the FDI World Dental Federation and its project partners.

All answers submitted are completely **anonymous**, and cannot be traced back to you by anyone, including your dentist. You may change your mind at any point if you decide you no longer wish to participate. If you have any further questions about the survey, please ask a member of your dental practice team.

We sincerely thank you for your participation in the OHO and contribution to improving oral health!

Annex C: Participant Consent Form

The following is the text of the participant consent form, which will appear on the mobile application before participants begin the questionnaire. Participants will need to select the option 'I accept' before proceeding with the survey.

Oral Health Observatory Project: Consent Form

Please read the following points and click on '*I Accept*' to confirm that you agree and are willing to complete the OHO questionnaire. If the participant is under 12 years old, a parent or guardian should complete this form on their behalf:

1. I confirm that I have received and read the OHO participant information sheet and have had chance to consider the information and ask questions.

2. I understand that my participation is voluntary and that I am free to withdraw at any time without giving any reason, without my medical care or legal rights being affected.

3. I understand that the information about me collected through this project will be stored anonymously by FDI World Dental Federation and its project partners, and used for research purposes.

4. I agree to take part in the study.

Please click on '*I Accept*' if you agree with the above statements and are ready to start the questionnaire.

References

1. Glick M, Williams DM, Kleinman DV, Vujicic M, Watt RG, Weyant RJ. A new definition for oral health developed by the FDI World Dental Federation opens the door to a universal definition of oral health. The Journal of the American Dental Association. 2016 Dec 1;147(12):915–7.

2. Oral Health and Well-Being in the United States: Data and Methods. USA, American Dental Association, Health Policy Institute, 2015 (http://www.ada.org/en/science-research/health-policy-institute/oral-health-and-well-being, accessed 23 February 2017).

3. Atchison KA, Dolan TA. Development of the Geriatric Oral Health Assessment Index. J Dent Educ 1990 54(11): 680–687.

4. Slade GD. Derivation and Validation of a Short-Form Oral Health Impact Profile. Community Dent Oral Epidemiol 1997 25(4): 284–290.

5. Adulyanon S, Sheiham A. In Slade GD (ed) Oral Impacts on Daily Performances in Measuring oral health and quality of life. pp. 151–160. Chapel Hill: University of North Carolina, 1997.

6. Avraham Zini et al. Validation of an Innovative Instrument of Positive Oral Health and Well-Being (POHW), Quality of Life Research. Qual Life Res 2016, 25(4): 847–858.

7. Peterson PE, Baez RJ, World Health Organization. WHO Oral Health Surveys: Basic Methods - 5th Edition (Monts: Présence Graphique, 2013): 111–118.

8. World Health Organization. World health statistics 2014. Geneva: World Health Organization; 2014.
